# Current practices in prevention, screening, and treatment of diabetes in kidney transplant recipients: European survey highlights from the ERA DESCARTES Working Group

**DOI:** 10.1093/ckj/sfae367

**Published:** 2024-12-10

**Authors:** Yassine Laghrib, Luuk Hilbrands, Gabriel C Oniscu, Marta Crespo, Ilaria Gandolfini, Christophe Mariat, Geir Mjøen, Mehmet Sukru Sever, Bruno Watschinger, Arzu Velioglu, Erol Demir, Eva Gavela Martinez, Annelies De Weerd, Ivana Dedinska, Maria Pippias, Annick Massart, Daniel Abramowicz, Johan Willem de Fijter, Christophe De Block, Rachel Hellemans

**Affiliations:** Department of Nephrology-Hypertension, Antwerp University Hospital, Edegem, Belgium; Faculty of Medicine & Health Sciences, Laboratory of Experimental Medicine and Paediatrics (LEMP), University of Antwerp, Wilrijk, Belgium; Department of Nephrology, Radboud University Medical Center, Nijmegen, the Netherlands; Transplant Division, Department of Clinical Science, Intervention and Technology, Karolinska Institute, Stockholm, Sweden; Department of Nephrology, Hospital del Mar Barcelona, Barcelona, Spain; Department of Medicine and Surgery, University of Parma, Parma, Italy; Nephrology Unit, University Hospital of Parma, Parma, Italy; Service de Néphrologie, Dialyse et Transplantation Rénale, Hôpital Nord, CHU de Saint-Etienne, France; Lovisenberg Hospital, Oslo Norway; Division of Nephrology, Department of Internal Medicine, Istanbul Faculty of Medicine, Istanbul, Türkiye; Department of Nephrology, Medical University of Vienna, Vienna, Austria; Marmara University, School of Medicine, Department of Nephrology, Istanbul, Türkiye; Transplant Immunology Research Centre of Excellence, Koç University Hospital, Istanbul, Türkiye; Nephrology Department, Hospital Peset, Valencia, Spain; Department of Internal Medicine, Erasmus MC Transplant Institute, University Medical Center Rotterdam, Rotterdam, Netherlands; Transplant Centre, University Hospital Martin, Martin, Slovakia; North Bristol NHS Trust, Renal Unit, Bristol, UK; Bristol Medical School: Population Health Sciences, University of Bristol, Bristol, UK; Department of Nephrology-Hypertension, Antwerp University Hospital, Edegem, Belgium; Faculty of Medicine & Health Sciences, Laboratory of Experimental Medicine and Paediatrics (LEMP), University of Antwerp, Wilrijk, Belgium; Department of Nephrology-Hypertension, Antwerp University Hospital, Edegem, Belgium; Faculty of Medicine & Health Sciences, Laboratory of Experimental Medicine and Paediatrics (LEMP), University of Antwerp, Wilrijk, Belgium; Department of Nephrology-Hypertension, Antwerp University Hospital, Edegem, Belgium; Faculty of Medicine & Health Sciences, Laboratory of Experimental Medicine and Paediatrics (LEMP), University of Antwerp, Wilrijk, Belgium; Faculty of Medicine & Health Sciences, Laboratory of Experimental Medicine and Paediatrics (LEMP), University of Antwerp, Wilrijk, Belgium; Endocrinology, Diabetology & Metabolism, Antwerp University Hospital, Edegem, Belgium; Department of Nephrology-Hypertension, Antwerp University Hospital, Edegem, Belgium; Faculty of Medicine & Health Sciences, Laboratory of Experimental Medicine and Paediatrics (LEMP), University of Antwerp, Wilrijk, Belgium

**Keywords:** kidney transplantation, prevention, PTDM, screening, transplant care

## Abstract

**Background:**

Although post-transplant diabetes mellitus (PTDM) is a common complication after kidney transplantation, there are few data on prevention, optimal screening, and treatment strategies.

**Methods:**

The European Renal Association's DESCARTES working group distributed a web-based survey to European transplant centres to gather information on risk assessment, screening procedures, and management practices for preventing and treating PTDM in kidney transplant recipients.

**Results:**

Answers were obtained from 121/241 transplant centres (50%) across 15 European countries. Screening practices for diabetes mellitus during the transplant work-up varied, with only 13% of centres using the recommended oral glucose tolerance test (OGTT) and 14% not screening at all. At transplantation, 19% of centres tailored the immunosuppressive regimen based on perceived PTDM risk, using strategies such as cyclosporin use or early steroid withdrawal. Fifty-two percent adopted strict glycaemic control with basal insulin in the first days post-transplant. Sixty-eight percent had defined screening protocols for early PTDM (45 days–6 months), primarily based on fasting glycaemia and/or HbA1c, while only a minority (7%) incorporated an OGTT. Changes in immunosuppression were considered by 41% in cases of early hyperglycaemia (<45 days) and by 58% in established PTDM (>45 days). Besides insulin therapy, dipeptidyl peptidase-4 (DPP4) inhibitors and metformin were most frequently used to manage early hyperglycaemia (<45 days) and PTDM (>45 days). The use of SGLT2 inhibitors and GLP-analogues increased >45 days post-transplantation.

**Conclusion:**

This European survey underscores the significant variation in PTDM prevention, screening, and treatment practices, emphasizing the imperative for more explicit guidance in approaching this complication.

## Graphical Abstract

**Graphical Abstract:**
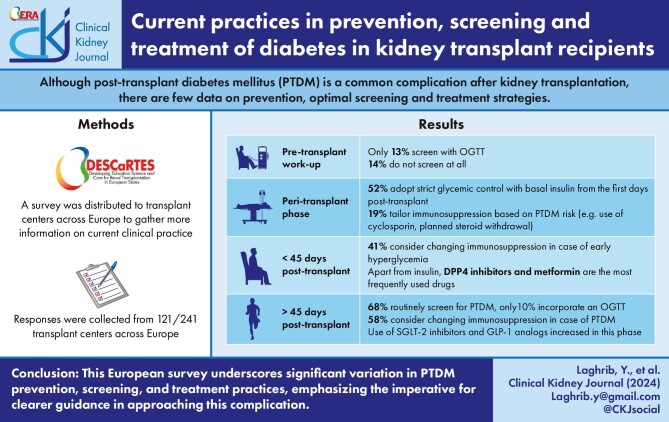

KEY LEARNING POINTS **What was known**:

- Post-transplant diabetes mellitus (PTDM) is diagnosed in 10%–40% kidney transplant recipients in the first post-transplant year. Diagnosing diabetes can be challenging in the pre-transplant and early post-transplant phase. An oral glucose tolerance test (OGTT) is the preferred screening test.
- The absence of universally validated predictive tools for identifying PTDM risk, alongside the lack of strong evidence on effective preventive measures (e.g. tailoring immunosuppression and tight glycaemic control with insulin therapy), or optimal treatment strategies, presents a significant hurdle in clinical practice.

**This study adds**:

- Only few transplant centres routinely perform an OGTT during the pre-transplant work-up, which may lead to an underestimation of diabetes prevalence in transplant candidates. Similarly, the underutilization of OGTT in the post-transplant setting probably contributes to an underestimation of PTDM.
- A standard assessment of PTDM risk is lacking, creating a gap in patient care and opportunities for early intervention.
- There exists hesitancy in prescribing new antidiabetic medications to kidney transplant recipients during the initial post-transplant period.

**Potential impact**:

- Rigorous screening for undiagnosed diabetes in kidney transplant candidates and recipients can facilitate tailored interventions, mitigating potential complications.
- Accurate, user-friendly PTDM risk prediction tools are needed to individualize the glucose monitoring intensity post-transplant and to drive research on preventive strategies for high-risk patients.
- Efforts should focus on identifying optimal treatments for PTDM, necessitating further research on the newer antidiabetic drugs in this population.

## INTRODUCTION

Post-transplant diabetes mellitus (PTDM) is a significant complication following kidney transplantation. In addition to conventional risk factors such as age, body mass index (BMI), and lifestyle, the administration of immunosuppressive agents can disturb glycaemic homeostasis, precipitating the onset of PTDM in 10% to 40% of kidney transplant recipients within the first year, with the highest incidence between 3 and 6 months post-transplantation [1–3]. PTDM is not only linked to higher graft failure rates but also to an increased incidence of cardiovascular events and infections, which are the leading causes of death among kidney transplant recipients [4]. In fact, a large US Renal Data registry study of >27 000 kidney transplant recipients from 1999 to 2002 found that both acute rejection and PTDM have a similar deleterious impact on long-term transplant survival [4]. A recent meta-analysis reported a 67% increase in all-cause mortality and a 35% increase in graft failure in patients with PTDM compared to nondiabetic patients [5].

Despite these worrisome data, current guidelines lack precision on PTDM prevention, screening, and management (Table 1), leading to considerable heterogeneity in clinical practice across transplant centres [6–10].

**Table 1:** Overview of current guidelines.

	KDIGO^6,7^	United Kingdom Renal Association and British Clinical Diabetologists^7^	Consensus Report 2024^9^
Pre-transplantation			
• Screening for diabetes	OGTT^6^	FG ± HbA1c High-risk patients should then go on to have OGTT to confirm diagnosis.	OGTT
• Repeat screening?	Not mentioned	Annual	Not mentioned
• Pre-transplant risk assessment	Indicated No specific tool mentioned^6^	Indicated e.g. Chakkera score	Indicated Clinical phenotypes or novel risk prediction models like polygenic risk scores can be used.
Transplantation (direct postoperative period)			
• Screening for diabetes/hyperglycaemia	Not mentioned	Afternoon glycaemia	Not mentioned
• Immunosuppression choice	Not mentioned	Based on immunological and hyperglycaemic risk	Irrespective of PTDM risk
• Other	Not mentioned		Lifestyle modification + exercise
Post-transplantation²			
• Screening for PTDM	FG, OGTT, and/or HbA1c^10^	OGTT is gold standard, HbA1c, FG	OGTT is gold standard
• Frequency of screening	at least weekly for 4 weeks, every 3 months for 1 year, and annually thereafter^10^	Not mentioned	10–13 weeks post-transplant (early PTDM), 1 year and onwards (late PTDM)
• Immunosuppression regarding PTDM	Consider modifying^10^	Adaptation of immunosuppression should be balanced against the risk of acute rejection	Irrespective of PTDM risk
• Treatment of PTDM	Not mentioned	Glycaemia <250 mg/dl (14 mmol/l) oral hyperglycaemic therapy, otherwise insulin	<45 days insulin and second, oral therapy >45 days lifestyle modification, oral treatment, and insulin therapy

FG = fasting glycaemia, HbA1c haemoglobin A1c, OGTT = oral glucose tolerance test, RG = random glycaemia (6,7,9,10 = references),

^6^KDIGO Transplant Candidate Guidelines 2020.

^7^Association of British Clinical Diabetologists and Renal Association guidelines on the detection and management of diabetes post solid organ transplantation 2021.

^9^International consensus on post-transplantation diabetes mellitus.

^10^KDIGO Recipients Guidelines 2009.

Until now, this presumed heterogeneity in management has remained unexplored. Hence, the survey was designed to gain a better understanding of current practices in the prevention and management of PTDM across Europe. Our primary objective was to raise awareness of this complication, assess clinical practices and pinpoint areas for improvement.

## MATERIALS AND METHODS

### Survey design

This brief 10-minute online survey investigated variations in daily practices among European kidney transplant centres concerning the screening, prevention, and treatment of PTDM. The survey comprised 25 main questions (with additional sub-questions for nine main questions). It was focused on four distinct stages: (i) pre-transplant work-up, (ii) the day of transplantation, (iii) early post-transplant phase (≤45 days), and (iv) beyond 45 days post-transplant. Tables 1 and 2 (also Supplemental S1) comprehensively overview all questions. Y.L., D.A., C.D.B., and R.H. collaboratively designed the initial draft of the survey. The survey was then refined based on feedback from all European Renal Association-DESCARTES working group board members. All members reached a consensus on the format and wording of the questions. The survey was transformed from a standard document into an online format using Qualitrics XM^®^.

**Table 2:** Screening for diabetes mellitus pre-transplantation, risk assessment for PTDM, and management of hyperglycaemia peri-transplantation.

**Pre-transplant work-up**			
1.‘Do you routinely screen for diabetes mellitus in the pretransplant work-up?’ Yes *n* = 104/121 (86%)			
If yes:			
1.1. ‘How do you routinely screen for diabetes mellitus in the pretransplant work-up?’ (more than one answer possible)			
• HbA1c	90/104 (87%)	• Random glycaemia	26/104 (25%)
• Fasting glycaemia	73/104 (70%)	• OGTT	14/104 (13%)
1.2. ‘Since the time between the pre-transplantation work-up and the actual transplantation can be several years, do you repeat the diabetes screening during the waiting period?’			
• Yes (at least) annually	59/104 (57%)		
• Sometimes	34/104 (33%)		
• No	11/104 (11%)		
2. ‘Do you have a specific weight-management program for obese transplant candidates?’			
Yes *n* = 64/121 (53%)			
If yes:			
2.1. ‘Which options are included in your weight-management program?’ (more than one answer possible)			
• Diet and intensive follow-up by a dietician	62/64 (97%)	• Exercise programme	28/64 (44%)
• Bariatric surgery	44/64 (69%)	• GLP-1 analogue	28/64 (44%)
3. ‘Do you record the family history of diabetes mellitus in the patient's medical file?’			
• Yes always	75/121 (62%)		
• Sometimes	37/121 (31%)		
• No	9/121 (7%)		
**Transplantation**			
4. ‘How do you routinely screen for pre-existing diabetes mellitus on the day of transplantation?’ (more than one answer possible)			
• HbA1c	73/121 (60%)		
• Random glycaemia	73/121 (60%)		
• Fasting glycaemia in both living and deceased donor recipients	52/121 (43%)		
• Fasting glycaemia only in living donor recipients	24/121 (20%)		
• OGTT only in living donor recipients	7/121 (6%)		
• OGTT in both living and deceased donor recipients	3/121 (2%)		
5. ‘Do you have a differentiated post-transplant management plan (e.g. choice of immunosuppression, intensity of glucose monitoring) in advance based on your perceived PTDM risk on the day of transplantation?’			
Yes *n* = 48/121 (40%)			
If yes:			
5.1. ‘Which items do you include in your risk assessment for PTDM on the day of transplantation?’ (more than one answer possible)			
Demographics		Laboratory values	
• Age	34/48 (71%)	• Triglyceridaemia	16/48 (33%)
• Family history	35/48 (73%)	• HbA1c	42/48 (88%)
• BMI	44/48 (92%)	• Fasting glycaemia	36/48 (75%)
• Waist circumference	10/48 (21%)	Models	
• Waist/hip ratio	4/48 (8%)	Chakkera model	2/48 (4%)
		Findrisc	1/48 (2%)
79% of the surveyed transplant centres use four or more predictors for their risk assessment.			
5.2. ‘Do you routinely consider a different immunosuppressive strategy on the day of transplantation in patients deemed at higher risk of PTDM?’ Yes *n* = 23/48 (48%)			
If yes:			
5.2.1. ‘Yes, I consider one or more alternative strategies:…’ (more than one answer possible)			
CNI regimen		Stop corticosteroids	
• CsA instead of Tac	12/23 (52%)	• First week	2/23 (9%)
• mTORi instead of CNI	1/23 (4%)	• 1 week–3 months	9/23 (39%)
• mTORi + lower dose of CNI	1/23 (4%)	• >3 months	7/23 (30%)
• Belatacept instead of CNI	1/23 (4%)		
CsA = cyclosporin, Tac = tacrolimus, mTORi = mammalian Target of Rapamycin inhibitor, CNI = calcineurin inhibitor			
6. ‘Do you routinely apply very tight glycaemic control during the early post-transplant period with the use of long-acting insulin therapy once the postoperative afternoon glucose value exceeds 140 mg***/***dL (7.8 mmol/L), targeting a pre-dinner glycemia of 110 mg/dl (6.1 mmol/L) post-transplant, such as advocated in the study by Schwaiger *et al.*?’			
• No	58/121 (48%)		
• Yes, but with slightly different glucose target levels than those advocated by Schwaiger *et al*. [1]	23/121 (19%)		
• Yes, in all patients	21/121 (17%)		
• Yes, in selected patients deemed at high risk of developing PTDM	19/121 (16%)		
7. ‘How long do you continue glucose day profile monitoring during hospitalization after transplantation?’			
• Routinely during the first 4–7 days (and continued in those who develop hyperglycaemia)	55/121 (46%)		
• Routinely, more than 7 days	41/121 (34%)		
• Routinely during the first 1–3 days (and continued in those who develop hyperglycaemia)	25/121 (21%)		

### Survey distribution

The DESCARTES board members established a steering committee overseeing this online survey. Each committee member distributed the survey link and a brief information letter via email to all kidney transplant centres for adult patients in their respective countries (11 countries). The targeted countries included Turkey (*n* = 44 centres), Italy (*n* = 39), Spain (*n* = 34), France (*n* = 32), UK (*n* = 24), Belgium (*n* = 7), Netherlands (*n* = 7), Sweden (*n* = 4), Slovakia (*n* = 4), Austria (*n* = 4), and Norway (*n* = 1). Furthermore, DESCARTES board members extended the survey to their network outside their own countries, reaching additional European countries (four countries), including Germany (*n* = 30), the Czech Republic (*n* = 6), Bosnia Herzegovina (*n* = 4), and Estonia (*n* = 1).

The survey link was actively distributed from April 2023 to September 2023. To ensure diversity and avoid duplication, each transplant centre was limited to one response, and the survey had to be completed by a nephrologist experienced in early post-transplant patient care. All participants were encouraged to register as survey contributors.

### Data analysis

**Figure 1:**
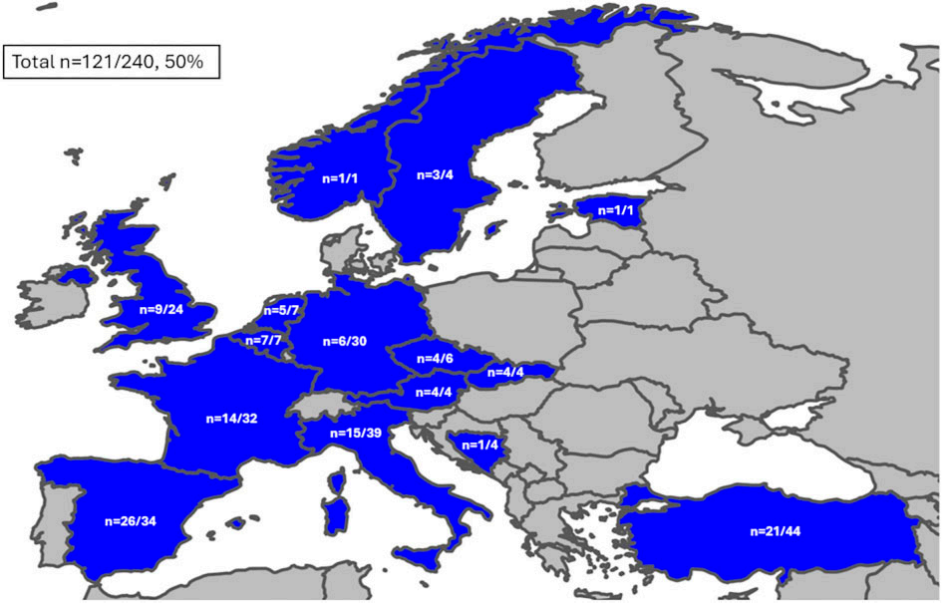
The survey's distribution across Europe with a response rate of 121/241 (50%). The graphical representation illustrates the response rates across various European countries, culminating in an overall response rate of 121 out of 241, equivalent to 50%. The response rates for individual countries are as follows: Turkey (21/44), Italy (15/39), Spain (26/34), France (14/32), Germany (6/30), UK (9/24), Belgium (7/7), Netherlands (5/7), Czechia Republic (4/6), Austria (4/4), Slovakia (4/4), Sweden (3/4), Bosnia Herzegovina (1/4), Estonia (1/1), and Norway (1/1).

The data were collected through Qualitrics XM®, an online survey tool. Descriptive statistics, portraying frequencies in percentage, were employed for data representation. IBM's SPSS version 29 facilitated the statistical analysis. The World map (Fig. 1) was generated using ggplot2 within R Studio version 2023.09.0 + 463. The data is presented in percentages when a question is posed to all surveyed centres. Alternatively, we use the format ‘number positive/number asked’ when the question is specifically directed to a subset of the surveyed centres.

## RESULTS

### General characteristics

**Figure 2:**
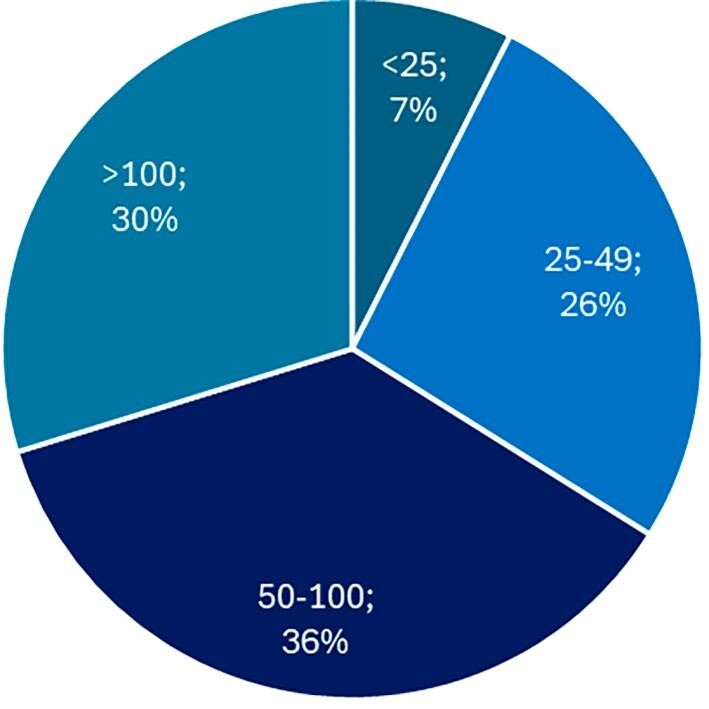
Distribution of transplant centre size. The graph illustrates the distribution based on centre size, revealing that 30% of centres performed >100 transplantations annually, 36% conducted between 50 and 100 transplantations annually, 27% carried out between 25 and 49 transplantations annually, and 7% managed <25 transplantations annually.

Data were collected from a comprehensive survey conducted across 241 transplant centres spanning 15 European countries (Fig. 1). Completed surveys were returned from 121 centres, leading to a response rate of 50%. Among the respondents, 95% had at least 5 years of experience in the early post-transplant field, and 93% of the responding centres performed 25 or more transplant procedures annually (Fig. 2).

### Pre-transplant work-up

During the pre-transplant work-up, 86% of transplant centres routinely screen to identify pre-existing diabetes mellitus. The methods employed for screening include primarily the use of HbA1c (87%) and fasting glycaemia (70%), followed by random glycaemia (25%), and oral glucose tolerance test (OGTT) (13%). Fifty-seven percent of transplant centres reported an annual repetition of these screening methods, with an additional 33% indicating repeated screening at some other timepoint and 10% never screened again. Regarding the identification and modification of risk factors for PTDM, it is noteworthy that ∼62% of transplant centres consistently document the family history of diabetes, while the remaining centres record it occasionally (31%) or never (7%). Fifty-three percent (*n* = 64) of centres have established weight-management programmes tailored to obese transplant candidates. These programmes involve dietary interventions and intensive monitoring by a dietician (62/64), the consideration of bariatric surgery (44/64), participation in structured exercise regimens (28/64), and the use of GLP-1 analogues (28/64) (as shown in Table 2).

### The day of transplantation

Screening tools commonly used to detect diabetes mellitus on the day of transplantation include HbA1c (60%) and random glycaemia (60%). Fasting glycaemia is more widely used in living donor transplants (63%) than deceased donor transplants (43%). The OGTT is rarely conducted on the day of transplantation (Table 2).

**Figure 3:**
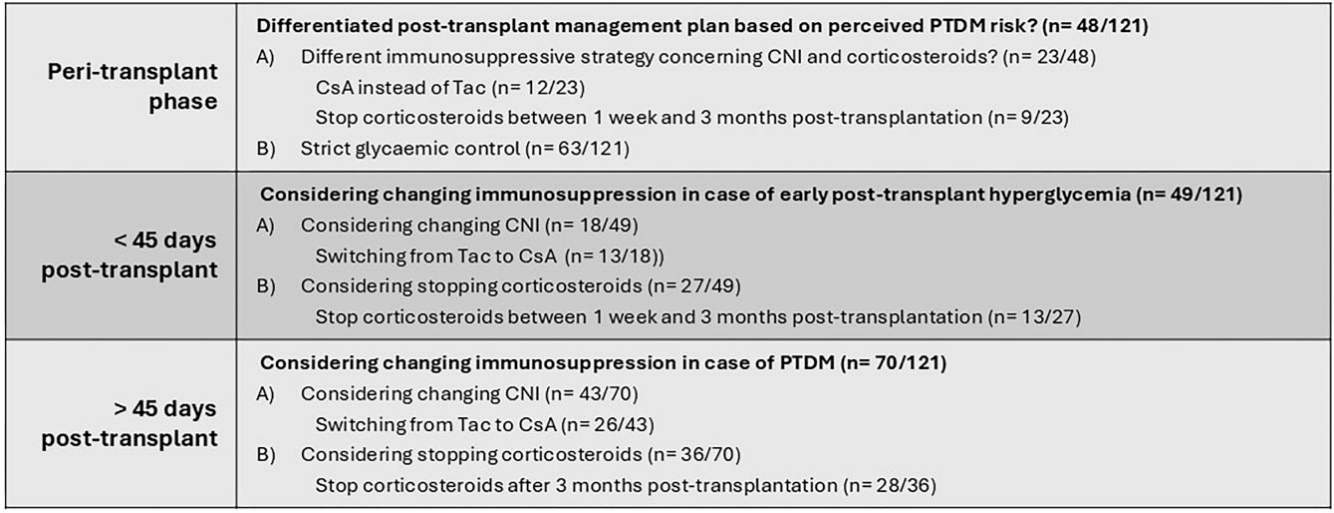
Overview of implemented strategies concerning prevention of PTDM. CNI = calcineurin inhibitor, CsA = cyclosporin, Tac = tacrolimus.

Notably, 40% (*n* = 48) of transplant centres have tailored post-transplant management plans for kidney transplant recipients deemed to have a higher risk of developing PTDM. These plans rely on various predictive indicators based on demographics, such as BMI (44/48), family history of diabetes mellitus (35/48), age (34/48), waist circumference (10/48), and waist-hip ratio (4/48). Additionally, biochemical markers, including HbA1c (42/48), fasting glycaemia (36/48), and fasting triglyceridaemia (16/48), are used. Only a minority of transplant centres use prediction models such as the Chakkera model [11] (2/48) and the Findrisc score (1/48) [12]. The differentiated post-transplant management plans include immunosuppression selection (23/48). The primary modification concerning immunosuppression involves a preference for cyclosporin over tacrolimus (12/23), followed by a semi-early cessation of corticosteroids between 1 week and 3 months post-transplantation (9/23). Seventeen percent of centres adhere to the protocol advocated by Schwaiger *et al.* [1] for all participants, while 16% reserve it for selected individuals deemed at increased risk of developing PTDM. Furthermore, an additional 19% adopt slightly modified glucose targets compared to those proposed by Schwaiger *et al.* (Table 2). An overview of the implemented strategies is also shown in Fig. 3.

### The early post-transplant phase (≤45 days)

Home glucose monitoring is implemented consistently in 7% of transplant centres, while 70% opt for a case-by-case approach, and 23% do not incorporate this practice. Additionally, 21% of transplant centres never refer individuals to an endocrinologist during the early post-transplant period. Conversely, for 27% of transplant centres, this referral is a standard procedure, while 52% make referrals on a case-by-case basis.

Concerning early post-transplant hyperglycaemia, 39% (*n* = 49) of transplant centres consider altering immunosuppression plans on a case-by-case basis. Among those contemplating changes, there is a preference for discontinuing corticosteroids (27/49), notably during the semi-early (13/27) to late post-transplant phases (12/27). Furthermore, 18/49 transplant centres consider modifying calcineurin inhibitors by transitioning from tacrolimus to cyclosporin (13/18) or reducing calcineurin inhibitor dosages (8/18).

Regarding treatment for early post-transplant hyperglycaemia, 30% exclusively use insulin. However, when considering therapies other than insulin, there is a preference hierarchy: DPP-4 inhibitors (61%), metformin (49%), sulfonylurea agents or glinides (45%), SGLT2 inhibitors (25%), and GLP-1 analogues (20%) (Table 3). An overview of the implemented strategies is also shown in Fig. 3.

**Table 3:** Post-transplant screening and management of hyperglycaemia.

**Early post-transplant phase <45 days**	
1. ‘Do you organize home blood glucose monitoring during the early post-transplant phase (≤45 days)?’	
• Yes, on a case-per-case basis	85/121 (70%)
• No	28/121 (23%)
• Yes, always	8/121 (7%)
2. ‘Do you refer patients with early post-transplant hyperglycaemia (≤45 days) to an endocrinologist?’	
• Yes, on a case-per case basis	63/121 (52%)
• Yes, always	33/121 (27%)
• No	25/121 (21%)
3. ‘Do you change immunosuppression in patients who develop early post-transplant hyperglycaemia (≤45 days)?’	
• No	72/121 (60%)
• Yes, on a case-per case basis	47/121 (39%)
• Yes, routinely in patients with standard risk for PTDM	2/121 (2%)
3.1. ‘Do you consider the withdrawal of corticosteroids in patients who develop early post-transplant hyper-glycemia (≤45 days)?’ Yes *n* = 27/49 (55%)	
If yes:	
3.1.1. ‘When do you consider the withdrawal of corticosteroids?’ (more than one answer possible)	
• First week	6/27 (22%)
• Week 1–3 months	13/27 (48%)
• >3 months	12/27 (44%)
3.2. ‘Do you consider changing, avoiding, or minimizing calcineurin inhibitors in patients who develop early post-transplant hyperglycaemia in the first 45 days?’ Yes *n* = 18/49 (37%)	
If yes:	
3.2.1. ‘What do you consider for patients who develop early post-transplant hyperglycaemia in the first 45 days and are on a standard regimen with calcineurin inhibitors and mycophenolate?’ (more than one answer possible)	
• Switch to CsA instead of Tac	13/18 (72%)
• Reduction CNI dose	8/18 (44%)
• Switch to mTORi + low-dose CNI	4/18 (22%)
• Switch to Belatacept instead of CNI	2/18 (11%)
• Switch to mTORi instead of CNI	1/18 (6%)
CsA = cyclosporin, Tac = tacrolimus, mTORi = mammalian Target of Rapamycin inhibitor, CNI = calcineurin inhibitor	
4. ‘Do you use antidiabetic drugs other than insulin in hyperglycaemic patients during the early post-transplant period (≤45 days)?’ Yes *n* = 85/121 (70%)	
4.1. ‘Which antidiabetic drugs other than insulin do you consider in the early post-transplant period (≤45 days)?’ (more than one answer possible)	
• DPP-4 inhibitors	52/85 (61%)
• Metformin	42/85 (49%)
• Sulfonylurea or glinides	38/85 (45%)
• SGLT2-inhibitors	21/85 (25%)
• GLP-1 analogues	17/85 (20%)
**Post-transplant phase >45 days**	
5. ‘Do you have a defined protocol to screen for PTDM between 45 days and six months?’ Yes *n* = 82/121 (68%)	
If yes:	
5.1. ‘What is included in your protocol to screen for PTDM between 45 days and six months?’ (more than one answer possible)	
• Fasting glycaemia	70/82 (85%)
• HbA1c	70/82 (85%)
• Home glucose measurements	19/82 (23%)
• Random glycaemia	19/82 (23%)
• OGTT	8/82 (10%)
6. ‘Do you have a defined protocol to screen for PTDM annually?’ Yes *n* = 90/121 (74%)	
If yes:	
6.1. ‘What is included in your protocol to screen for PTDM annually?’ (more than one answer possible)	
• Fasting glycaemia	85/90 (94%)
• HbA1c	75/90 (83%)
• Random glycaemia	19/90 (21%)
• Home glucose measurements	13/90 (14%)
• OGTT	5/90 (6%)
7. ‘Do you refer patients diagnosed with PTDM after 45 days to an endocrinologist?’	
• Yes, on a case-per-case basis	58/121 (48%)
• Yes, this is the standard procedure	48/121 (40%)
• No	15/121 (12%)
8. ‘Do you change immunosuppression in patients diagnosed with PTDM after 45 days post-transplantation?’	
• Yes, on a case-per-case basis	69/121 (57%)
• No	51/121 (42%)
• Yes, this is the standard procedure	1/121 (1%)
If yes:	
8.1. ‘Do you consider the withdrawal of corticosteroids after 45 days post-transplantation?’ Yes *n* = 36/70 (51%), when: (more than one answer possible)	
Stop corticosteroids	
• Steroidal withdrawal after 3 months	28/36 (78%)
• Intermediate-early steroid withdrawal (between 6 weeks–3 months post-transplant)	14/36 (39%)
8.2. ‘Do you consider changing, avoiding, or minimizing calcineurin inhibitors in patients who develop PTDM after 45 days post-transplantation?’ Yes *n* = 43/70 (61%) (more than one answer possible)	
CNI regimen	
• Switch to CsA instead of Tac	26/43 (60%)
• Reduction CNI dose	24/43 (56%)
• Switch to mTOR + low-dose CNI	13/43 (30%)
• Switch to mTOR instead of CNI	8/43 (19%)
• Switch to Belatacept instead of CNI	3/43 (7%)
CsA = cyclosporin, Tac = tacrolimus, mTORi = mammalian Target of Rapamycin inhibitor, CNI = calcineurin inhibitor	
9. ‘Which antidiabetic drugs other than insulin do you consider in patients who have developed PTDM (after 45 days post-transplant)?’ (more than one answer possible)	
• Metformin	52/70 (74%)
• DPP-4 inhibitors	49/70 (70%)
• SGLT2-inhibitors	48/70 (69%)
• GLP-1 analogues	45/70 (64%)
• Sulfonylurea or glinides	28/70 (40%)

### Beyond 45 days post-transplant

At 68% (*n* = 82) of transplant centres, screening for PTDM is routinely conducted between 45 days and 6 months post-transplantation. This screening involves fasting glycaemia (70/82), HbA1c (70/82), home glucose measurements (19/82), random glycaemia (19/82), and/or OGTT (8/82). Subsequently, 74% (*n* = 90) of the transplant centres have established protocols for annual PTDM screenings. The preferred screening methods for the annual screening include HbA1c (85/90) as well as fasting glycaemia (75/90), random glycaemia (19/90), home glucose measurements (13/90), and OGTT (5/90).

When dealing with patients diagnosed with PTDM, 40% of transplant centres routinely refer them to an endocrinologist. In comparison, 48% do so on a case-by-case basis, and 12% do not refer patients. Among PTDM cases, 42% of transplant centres do not consider altering immunosuppression, while 58% (*n* = 70) consider changes (on a case-by-case basis). Alterations in calcineurin inhibitor treatments (43/70) are preferred, such as transitioning from tacrolimus to cyclosporin (26/43), reducing calcineurin inhibitor dosage (24/43), combining low-dose calcineurin inhibitors with mTOR inhibitors (13/43), or using mTOR inhibitors instead of calcineurin inhibitors (8/43). A small number (3/43) apply a switch from calcineurin inhibitors to belatacept. Additionally, 36/70 contemplate discontinuing corticosteroids, particularly after 3 months post-transplantation (28/36) and between 6 weeks and 3 months post-transplant (14/36).

Where antidiabetic treatment other than insulin is used, transplant centres prescribe metformin (52/70), DPP-4 inhibitors (49/70), SGLT2 inhibitors (48/70), GLP1 analogues (45/70), and sulfonylurea agents or glinides (28/70).

Half of the respondents surveyed confirmed having data regarding the incidence of PTDM in their centre, with rates ranging from 5% to 37% (mostly between 20% and 25%). When the respondents had no precise knowledge of the incidence of PTDM in their centre, the estimates ranged from 1% to 40% (mostly between 20% and 25%), as illustrated in the Supplemental S2 additional figures. There was no different management approach between large and small centres (Supplemental S3).

## DISCUSSION

Our study confirmed disparities in the screening and management of PTDM among various European transplant centres. This variability is expected, given the paucity of data, the lack of solid evidence, and the poorly defined standards of care in the guidelines published by the Kidney Disease Improving Global Outcomes (KDIGO) and the American Diabetes Association (ADA) [10, 13, 14].

The 2020 KDIGO guidelines, among others, recommend employing an OGTT in the pre-transplant work-up for several reasons [6, 8, 9, 15]; in individuals with end-stage kidney disease, fasting glycaemia is a less reliable predictor of overall glycaemia control because of reduced renal gluconeogenesis, and HbA1c is often ‘false low’ due to a shorter erythrocyte life span [16–21]. Several studies have demonstrated that the accuracy of HbA1c as a marker for glycaemic control diminishes compared to fasting or random glycaemia in patients with known diabetes as CKD progresses [22–27]. Notably, a recent single-centre study demonstrated that patients with an HbA1c level from as low as ≥5.3% on the day of transplantation have a >35% risk of developing PTDM within 3 months, which also suggests that HbA1c measurements in the pre-transplant setting may be inaccurately low [28]. Despite current recommendations for pre-transplant diabetes screening using an OGTT, our survey reveals that 14% of transplant centres skip screening for diabetes mellitus, and among those who screen, the primary methods include HbA1c (87%) and fasting glycaemia (70%). Of note, only 13% of surveyed transplant centres include an OGTT in their pre-transplant work-up. Factors such as additional hospital visits, fasting requirements, and potential disruption to peritoneal dialysis schedules may contribute to the centre's hesitancy.

The pre-transplant phase also represents a critical period for addressing modifiable risk factors associated with PTDM, notably obesity. However, weight loss often proves to be a significant challenge for individuals with end-stage kidney disease due to dietary restrictions and limited physical mobility [29]. Hence, professional support would benefit these subjects [30]. Our survey findings revealed that 53% of centres have implemented weight-management programmes tailored to obese transplant candidates, primarily involving dietary counselling by dietitians and considering options such as bariatric surgery or GLP-1 analogues. Notably, only 23% of surveyed centres offer structured exercise programmes for these patients, indicating a potential area for improvement.

On the day of transplantation, only 40% of the surveyed transplant centres estimate the PTDM risk to tailor the post-transplant management plan accordingly (e.g. by implementing more intensive glucose monitoring or adapting immunosuppressive therapy in high-risk patients): only very few use existing prediction models such as the Findrisc and Chakkera scores [11, 12]. The Findrisc score, including eight variables, lacks validation in CKD, where certain factors, such as low daily vegetable consumption (reflecting adherence to a low-potassium diet) and a history of hypertension, may exhibit diminished discriminatory capacity [11, 12, 31]. Meanwhile, the Chakkera score, based on seven variables to predict PTDM in kidney transplant recipients, has also not been implemented in clinical practice or research. This is potentially due to its reliance on fasting laboratory parameters (glycaemia and triglycerides), which are more difficult to obtain and subjective criteria (the intention to maintain corticosteroid therapy beyond 1 week post-transplantation) [11].

Only 19% of surveyed centres routinely adjust immunosuppressive therapy from the day of transplantation based on perceived PTDM risk, primarily by opting for cyclosporine over tacrolimus or by implementing (semi)-early steroid withdrawal between 1 week and 3 months post-transplantation. The reluctance to modify immunosuppression to mitigate PTDM risk probably stems from concerns regarding increased rejection rates. While consistent evidence favours cyclosporine's lower diabetes induction than tacrolimus [32–34], debates continue over its potentially higher rejection risk. This is partly due to dated comparative studies, which challenge extrapolation to contemporary practice. Attention to concomitant immunosuppression, such as ensuring adequate mycophenolate dosing, is crucial. Early studies on steroid withdrawal yielded mixed findings regarding PTDM prevention and rejection risk [35]. However, recent trials have provided reassurance in this regard. The HARMONY trial, a large multicentre randomized controlled trial (*n* = 587), demonstrated that rapid steroid withdrawal on day 8, coupled with either interleukin 2 receptor agonist (IL2-RA) or anti-thymocyte globulin induction and tacrolimus/mycophenolate maintenance therapy, reduced PTDM incidence by 40% at 1 year without increasing rejection risk compared to maintaining steroids [2]. The initial decline in incidence was not counterbalanced by a subsequent rise over the 5-year follow-up period [36]. Similarly, the ADVANCE trial, a randomized controlled trial (*n* = 1081) involving two steroid minimization strategies (steroid withdrawal at day 10 versus avoidance), with both arms receiving IL2-RA induction, tacrolimus, and mycophenolate therapy, reported comparable 6-month biopsy-proven acute rejection rates to the HARMONY trial and other contemporary cohorts [37–39]. The arm that kept steroids until day 10 had significantly less biopsy-proven rejection (9% vs. 14%), and PTDM incidences were low (17.4% vs 16.6%, NS). It is worth noting, however, that both trials were conducted in a standard immunological risk European population (first or second kidney transplants, with a panel reactive antibody level of ≤30% and no pre-transplant donor-specific antibodies), limiting their findings' generalizability. Until accurate predictors for rejection risk and PTDM incidence become available, making personalized decisions regarding the optimal immunosuppressive regimen will continue to pose challenges.

Our survey revealed that centres are more inclined to adjust immunosuppression later in the post-transplant phase, particularly when early hyperglycaemia persists during the first 45 days (reported in 41% of centres) or when PTDM is formally diagnosed (after 45 days, reported in 43% of centres). In 26 out of 121 surveyed centres, consideration is given to switching from tacrolimus to cyclosporine, a strategy supported by recent evidence: a randomized controlled trial involving 80 stable kidney transplant recipients with PTDM demonstrated that such a switch to cyclosporin led to improved glucose metabolism, and reversal of diabetes in significantly more patients during the first year compared to those who continued on tacrolimus (34% versus 10%, *P* = .01) without increasing the risk of acute rejection [40]. However, the efficacy of (semi-) late withdrawal of steroids, an option considered by 13 out of 121 transplant centres in case PTDM develops, in reversing diabetes remains a topic of debate.

Many transplant centres spend extra efforts to detect and rigorously manage early post-transplant hyperglycaemia. Thirty-four percent monitor daily glucose profiles for >1 week in all patients during hospitalization, while 77% conduct home monitoring on a case-by-case basis. This home-based monitoring approach may prove beneficial, as traditional outpatient hospital visits in the morning often overlook the peak glycaemic levels induced by corticosteroids in the afternoon or evening. Interestingly, 52% of respondents indicated initiating treatment with basal insulin for early hyperglycaemia once afternoon glucose levels surpass 140 mg/dl (7.8 mmol/l). This approach, leading to excellent glycaemic control, may consequently serve as a preventive measure against the development of PTDM [41, 42]. Indeed, a pilot trial conducted by Hecking *et al.* [41] demonstrated that such stringent glycaemic control with intermediate-acting insulin reduced the odds of PTDM by an impressive 73% at 1-year post-transplant, with the authors attributing this effect to enhanced preservation of beta cell function. However, a subsequent multicentre trial by the same group failed to replicate this effect [1]. Arguably, protocol violations, imbalances between the groups, and lower baseline risk of PTDM (e.g. due to lower steroid doses) may have attenuated any potential benefits. Nonetheless, the hypothesis that strict glycaemic control could mitigate glucotoxicity and prevent subsequent beta cell exhaustion continues to captivate researchers, with ongoing trials exploring the potential of DPP4-inhibitors and metformin post-transplant to prevent PTDM (NCT05240274 and NCT02849899).

Our survey also showed that, besides insulin, which is recommended by the recent consensus guidelines, centres show a preference for using DPP-4 inhibitors (61%), metformin (49%), and sulfonylureas/glinides (45%) over newer agents such as SGLT2 inhibitors (25%) and GLP-1 analogues (20%) to treat early post-transplant hyperglycaemia. In comparison, the use of these newer agents increases with time after transplantation. So far, nephrologists may exhibit reluctance toward SGLT2 inhibitors due to concerns about their potential impact on hemodynamic, low glucose-lowering efficacy with low glomerular filtration rate, and the potential risk of genito-urinary tract infections. At the same time, using GLP-1 analogues may raise concerns regarding gastrointestinal side effects. Evidence regarding the safety and efficacy of these drugs in the early post-transplant phase remains limited, as the first large studies with these newer agents did not include participants in this setting. Still, ongoing studies hold promise for elucidating these aspects. [NCT05702931 (Sema-RTX), NCT04965935 (SGLT2i), NCT03642184 (SGLT2i), NCT03113110 (SGLT2i)]

While the surveyed centres acknowledged the high incidence of PTDM during the initial months post-transplantation, only 6% utilized an OGTT for diagnosis, potentially leading to overlooking a considerable number of PTDM cases within the first 3 months post-transplantation [43, 44]. For example, a Norwegian cohort study (*n* = 1619) reported that 41% of PTDM diagnoses would have been missed if relying solely on fasting glycaemia and HbA1c in kidney transplant recipients without manifest PTDM (defined as individuals who develop PTDM before the clinical assessment) at 10 weeks post-transplant [44]. Once again, the logistical challenges associated with conducting an OGTT may serve as a significant deterrent, alongside scepticism regarding its added value compared to HbA1c, as PTDM cases identified solely through OGTT were reported to be more likely to regress to pre-diabetes with time [45]. However, a retrospective single-centre study has shown that even individuals diagnosed with PTDM solely through OGTT exhibit an elevated risk of mortality [46]. To gain greater confidence in the necessity of an OGTT and its optimal timing, further research is needed to ascertain its impact on patient clinical outcomes. After the initial year post-transplant, evidence indicates that HbA1c becomes more reliable for diagnosing PTDM [47, 48], a practice adopted by 70% of the surveyed centres.

This survey is the first to explore the prevention, screening, and management of PTDM in kidney transplant recipients. We gathered responses from a diverse group of transplant nephrologists across 15 European countries, ensuring a thorough understanding of practices on a continental scale. The 50% response rate suggests effective distribution and user-friendly survey completion. It adds credibility to the idea that our survey accurately reflects current clinical practices related to PTDM in European healthcare. The substantial response rate also emphasizes the survey's practical relevance, making it a valuable tool for understanding and potentially influencing real-world clinical practices related to PTDM.

There are several limitations to consider when interpreting the results of this survey. First, our survey includes countries in Europe selected by DESCARTES board members from the same country (*n* = 11) and additional countries belonging to their personal network (*n* = 4). As a result, the conclusions drawn from this study may not be directly applicable to other regions. Second, there is a possibility that respondents reported their ‘ideal’ behaviour rather than their typical practices. This tendency to exhibit idealized behaviour is common in self-reported surveys and might have influenced the accuracy of the reported data. We tried to minimize this by ensuring that responses could not be linked to individual centres and by allowing centres to respond anonymously (*n* = 10). Third, the survey did not distinguish between individual and collaborative team-based approaches in the follow-up care of kidney transplant recipients. Fourth, we received responses from only 50% of the contacted centres, potentially introducing a selection bias. The centres that responded may have a higher interest in PTDM, potentially influencing the care provided. However, since our survey revealed significant variations in reported practices that often diverged from guideline recommendations, the impact of this selection bias may be limited. Moreover, we gathered responses from a diverse mix of large, medium, and small transplant centres and found no trend indicating different behaviour based on centre size. From a statistical perspective, our sample of 121 out of 241 transplant centres yields a maximal margin of error of 6% at a 95% confidence level. This means our findings are reasonably accurate within this margin, allowing us to make reliable statements about the broader population despite inherent uncertainties. Last, to encourage higher response rates, we opted for a concise and straightforward survey design. However, this limited our ability to explore the underlying reasons behind the centres' policies, which could have offered deeper insights into their perspectives and concerns. In summary, through this survey, we have gained valuable insights into the current practices regarding the prevention, screening, and treatment of PTDM across 15 European countries. Significant diversity exists among centres. While some sensible measures, such as pre-transplant screening for (pre-)diabetes using an OGTT or providing multidisciplinary support for obese patients to facilitate weight loss, are recommended by authoritative guidelines (albeit based on low evidence), they are not consistently implemented in daily practice. The limited evidence and guidance on the optimal prevention, screening, and management of dysglycaemia during the post-transplant phase result in each centre adopting its own approach. Finally, nephrologists remain hesitant about incorporating newer antidiabetic medications such as GLP-1 analogues and SGLT2 inhibitors into the early post-transplant regimen, probably due to the limited evidence available concerning their safety and efficacy in this particular population. We hope this study's findings will raise awareness about this complication and trigger initiatives within the PTDM domain, driving improvements and standardization in screening and prevention practices.

## LIST OF CONTRIBUTORS

Respondents were invited to provide their credentials at the conclusion of the survey to be acknowledged as contributors. Those who opted not to share their credentials are consequently excluded from this list.

Serpil Muge Deger, Dokuz Eylul University, Faculty of Medicine, Department of Internal Medicine, Divison of Nephrology; İzmir Umut Kasapoglu, University of Health Sciences Bakirkoy Dr Sadi Konuk Training and Research Hospital, İstanbul; Başak Boynueğri, İstanbul Haydarpaşa Numune Hospital; Başar Aykent, Sisli, Hamidiye Etfal Training and Research Hospital; Ebru Sevinc ok, Kent Hastanesi, Elif Ari, Kartal Training Hospital; Ercan Turkmen, Ondokuz Mayis University, Samsun; Erhan Tatar, University of Health Sciences; İzmir Bozyaka, Training and Research Hospital, İzmir; Hamad Dheir, Sakarya University Faculty of Medicine; Havva Asuman Yavuz, Medicalpark Hospital; Hüseyin Töz, Medical Point Hospital, Izmir University of Economics, Izmir; Meltem Gursu, Bezmialem vakif university, faculty of medicine; Özant Helvacı, Gazi University Faculty of Medicine; Saime Paydas, Acıbadem Hospital Adana; Serkan Bakirdogen, Canakkale Onsekiz Mart University School of Medicine Department of Internal Medicine Division of Nephrology; Sultan Ozkurt, Department of Nephrology, Eskisehir Osmangazi University Faculty of Medicine, Eskisehir; Dilek Barutcu Atas, Marmara University School of Medicine, Department of Internal Medicine, Division of Nephrology, Istanbul, Turkey; Alessandra Panarese, University of L'Aquila; Andrea Ranghino, SOD Nefrologia, Dialisi e Trapianto Rene-AOU delle Marche; Francesco Perna, Nefrologia e Dialisi, Ospedale di Circolo e Fondazione Macchi di Varese, ASST dei Sette Laghi; Gaetano La Manna, Nephrology, Dialysis and Renal Transplant Unit, IRCCS-Azienda Ospedaliero-Universitaria di Bologna; Marilù Bartiromo, Nephrology, Dialysis and Transplant Unit, Careggi University Hospital, Florence; Papalia Teresa, Nephrology, dialysis and transplant unit, Riccardo Nappi, Nephrology, Dialysis and Transplant Unit, S. Maria della Misericordia Hospital; Udine Rosa Carrano, Azienda Ospedaliera Universitaria Federico II Napoli; Vincenzo Cantaluppi, Nephrology and Kidney Transplantation Unit, University of Piemonte Orientale (UPO); Alessandra Panarese, University of L'Aquila; Eleonora Calcaterra, ASST Settelaghi Varese; Rossana Caldara, San Raffaele Scientific Institute, Regenerative Medicine and Transplantation Milan; Alex Gutierrez-Dalmau, Department of Nephrology, Hospital Universitario Miguel Servet, IIS Aragón. Zaragoza; Ana Ramos Verde, Fundación Jiménez Díaz, Madrid; Ana Vila, Hospital germans trias i pujol; Carme Facundo Molas, Fundació Puigvert, Barcelona; Cristina Galeano Álvarez, Servicio de Nefrología. Hospital Universitario Ramón y Cajal Madrid; David Ramos, Hospital General Universitario de Castellón. Castellón de La Plana; Emilio Rodrigo, University Hospital Marqués de Valdecilla/Idival; Ingrid Auyanet, Hospital Universitario Insular de Gran Canaria; Inmaculada Lorenzo González, Complejo Hospitalario Universitario Albacete; Marco Montomoli, Hospital Clínico Universitario de Valencia; Maria Jose Torres Sanchez, Nefrologia, Hospital Universitario Virgen de las Nieves. Granada; Miguel Ángel Muñoz Cepeda, Renal transplant unit. University Hospital Toledo; Mònica Pérez Mir, Transplant Unit, Nephrology department, Fundació Puigvert, Barcelona; Sofia Zarraga, Chief of nephrology service of hospital Universitario Cruces; Ana M. González Rinne, Hospital Universitario de Canarias, Tenerife; Auxiliadora Mazuecos, Department of Nephrology. Hospital Universitario Puerta del Mar. 11009 Cadiz; Isabel Beneyto Castelló, Nefrología. H.U. La Fe. Valencia; Constantino Fernandez Rivera, Nephrology Department, Complexo Hospitalario Universitario A Coruña; Francesc Moreso, Nephrology Department. Hospital Universitari Vall d'Hebron. Barcelona; Guadalupe Tabernero Fernández, Transplant Nephrologist. Hospital Universitario de Salamanca; María Luisa Rodríguez Ferrero, S°Nefrología. H.U. Gregorio Marañón Madrid; Nuria Montero, Hospital Universitari de Bellvitge; Paloma L Martin-Moreno, Department of Nephrology, Clinica Universidad de Navarra Pamplona; Lionel Couzi, Néphrologie–Transplantation–Dialyse–Aphérèses, CHU Bordeaux; Sophie Girerd, Department of Nephrology, University Hospital of Nancy; Arwa Jalal Eddine, Department of Nephrology, Foch Hospital, Suresnes; R. Snanoudj, Nephrology department, Bicetre Hospital; Claire Tinel, Dijon University Hospital; Colosio, CHU Reims; Arnaud Del Bello, CHU de Toulouse; Gabriel Choukroun, CHU Amiens Picardie; Léonard Golbin, Service de Néphrologie, Dialyse et Transplantation rénale, Hopital Pontchaillou, CHU Rennes; Noble, CHU Grenoble-Alpes; Pernin, CHU Lapeyronie, Montpellier; Provot, Lille University Hospital; Klemens Budde, Charité Universitätsmedizin Berlin; Martina Guthoff, Dept. of Diabetology, Endocrinology, Nephrology, University of Tübingen, Tübingen; Anja Mühlfeld, Uniklinik RWTH Aachen; Thomas Rath, Westpfalz-Klinikum Kaiserslautern, Dep.of Nephrology and Transplantation Medicine; Martin Nitschke, University of Luebeck, Transplant Center; Uwe Heemann, Nephrology, Technical University Munich; Adnan Sharif, University Hospitals Birmingham; Daniel Stewart, Queen Alexandra Hospital, Portsmouth; Conor Byrne, Barts Health NHS Trust; Rachel Davison, South Tyneside and Sunderland NHS Foundation Trust; Joyce Popoola, St George's Hospitals NHS Foundation Trust; Paul Phelan, Royal Infirmary of Edinburgh; Pramod Nagaraja, Cardiff Transplant Unit, University Hospital of Wales, Cardiff; Steven Van Laecke, Ghent University Hospital; Concetta Catalano, Nephrology, Dialysis and Kidney Transplantation Unit, Erasme Hospital (Université libre de Bruxelles); Laurent Weekers CHU Liege; Lissa Pipeleers, Dpt Nephrology, UZ Brussel; Maarten Naesens, KU Leuven; Nadaa Kanaan, Saint-Luc Brussel; Raphael Duivenvoorden, Radboud University Medical Center; Aiko De Vries, Leiden University Medical; Ondrej Viklicky, Institute for Clinical and Experimental Medicine, Prague; Zdenek Lys, Department of Internal medicine and Cardiology, University Hospital Ostrava; Karel Krejčí, Deputy Head of Teaching, Head of the Nephrology Department, University Hospital and Faculty of Medicine and Dentistry, Palacký University I.P. Pavlova; Tomas Reischig, Dept. of Internal Medicine I, Charles University Teaching Hospital, Pilsen; Michael Rudnicki, Medical University Innsbruck; Kathrin Eller, Medical University of Graz, Division of Nephrology; Carin Wallquist, Dept. Nephrology, Skane Univ. Hospital Malmo; Jessica Smolander, Karolinska University Hospital; Bengt von Zur-Muehlen, Dept. of TransplantS, Uppsala University Hospital; Igor Gaľa, TC Kosice; Matej Vnučák, Transplant Center Martin; Tatiana Baltesová, Transplant department UNLP Kosice; Zuzana Zilinska, Univerzitná nemocnica Bratislava; Mirna Aleckovic-Halilovic, University Clinical Center Tuzla, Bosnia and Herzegovina; Külli Kõlvald, Tartu University Hospita Estonia; Trond Geir Jenssen, University Hospital of Oslo Rikshospitalet.

## Supplementary Material

sfae367_Supplemental_Files

## Data Availability

The article's data will be shared with the corresponding author at a reasonable request.

## Notes

Y.L., D.A., J.W.D.F., R.H., A.M., B.W., I.D., M.S.S., E.D., L.H., C.M., and M.C. have nothing to declare. E.G. received payment or honoraria from Valencian Society of Nephrology and Spanish Society of Nephrology, support for attending meetings from ERA EDTA Congress Milan 2023, Spanish Society of Nephrology Congress, 2022–2023 and Spanish Society of Transplantation Congress 2022. A.D.W. received grants from the Dutch Kidney Foundation, consulting fees from HANSA Biopharma, support for attending meetings from HANSA Biopharma and participation on a Data Safety Monitoring Board or Advisory Board for Regeneron. C.D.B. received consulting fees from Abbott, AstraZeneca, Boehringer Ingelheim, Eli Lilly and Company, Indigo Diabetes, Insulet, Medtronic, Novo Nordisk, and Roche and payment or honoraria from Abbott, Eli Lilly and Company, Insulet, Medtronic, Novo Nordisk. G.M. reported having a leadership or fiduciary role in ESOT. I.G. received consulting fees from Atheneum. M.P. reported having a leadership or fiduciary role in the International Society of Nephrology Emerging Leaders programme Cohort 2 (now completed). A.V. received payment or honoraria from Alexion. G.O. received grants from the Medical Research Council, ALF Medical Sweden, payment or honoraria from Asian Hepatobiliary Week, support for attending meetings from Aferetica Srl, participation on a Data Safety Monitoring Board or Advisory Board in DeFat trial, and leadership in ESOT.

## References

[1] Schwaiger E, Krenn S, Kurnikowski A et al. Early postoperative basal insulin therapy versus standard of care for the prevention of diabetes mellitus after kidney transplantation: a multicenter randomized trial. J Am Soc Nephrol 2021;32:2083–98. 10.1681/ASN.2021010127 34330770 PMC8455276

[2] Thomusch O, Wiesener M, Opgenoorth M et al. Rabbit-ATG or basiliximab induction for rapid steroid withdrawal after renal transplantation (Harmony): an open-label, multicentre, randomised controlled trial. Lancet North Am Ed 2016;388:3006–16. 10.1016/S0140-6736(16)32187-0 27871759

[3] Rodríguez-Rodríguez AE, Porrini E, Hornum M et al. Post-transplant diabetes mellitus and prediabetes in renal transplant recipients: an update. Nephron 2021;145:317–29. 10.1159/000514288 33902027

[4] Cole EH, Johnston O, Rose CL et al. Impact of acute rejection and new-onset diabetes on long-term transplant graft and patient survival. Clin J Am Soc Nephrol 2008;3:814–21. 10.2215/CJN.04681107 18322046 PMC2386715

[5] Lin H, Yan J, Yuan L et al. Impact of diabetes mellitus developing after kidney transplantation on patient mortality and graft survival: a meta-analysis of adjusted data. Diabetol Metabol Synd 2021;13. 10.1186/s13098-021-00742-4 PMC8557540 34717725

[6] Chadban SJ, Ahn C, Axelrod DA et al. KDIGO clinical practice guideline on the evaluation and management of candidates for kidney transplantation. Transplantation 2020;104:S11–S103. 10.1097/TP.0000000000003136 32301874

[7] Chowdhury TA, Wahba M, Mallik R et al. Association of British Clinical Diabetologist and Renal Association guidelines on the detection and management of diabetes post solid organ transplantation. (March 2022; date last accessed). 10.1111/dme.14523 33434362

[8] Sharif A, Hecking M, de Vries APJ et al. Proceedings from an international consensus meeting on posttransplantation diabetes mellitus: recommendations and future directions. Am J Transplant 2014;14:1992–2000. 10.1111/ajt.12850 25307034 PMC4374739

[9] Sharif A, Chakkera H, de Vries APJ et al. International consensus on post-transplantation diabetes mellitus. Nephrol Dial Transplant 2024;39:531–49. 10.1093/ndt/gfad258 38171510 PMC11024828

[10] Kasiske BL, Zeier MG, Chapman JR et al. KDIGO clinical practice guideline for the care of kidney transplant recipients: a summary. Kidney Int 2010;77:299–311. 10.1038/ki.2009.377 19847156

[11] Chakkera HA, Weil EJ, Swanson CM et al. Pretransplant risk score for new-onset diabetes after kidney transplantation. Diabetes Care 2011;34:2141–5. 10.2337/dc11-0752 21949218 PMC3177751

[12] LindströM J, Tuomilehto J. The diabetes risk score. Diabetes Care 2003;26:725–31. 10.2337/diacare.26.3.725 12610029

[13] Chadban SJ, Ahn C, Axelrod DA et al. Summary of the Kidney Disease: Improving Global Outcomes (KDIGO) clinical practice guideline on the evaluation and management of candidates for kidney transplantation. Transplantation 2020;104:708–14. 10.1097/TP.0000000000003137 32224812 PMC7147399

[14] ElSayed NA, Aleppo G, Aroda VR et al. 2. Classification and diagnosis of diabetes: standards of care in diabetes-2023. Diabetes Care 2023;46:S19–S40. 10.2337/dc23-S002 36507649 PMC9810477

[15] Malik RF, Jia Y, Mansour SG et al. Post-transplant diabetes mellitus in kidney transplant recipients: a multi-center study. Kidney360 2021;2:1296–1307. 10.34067/KID.0000862021 35369651 PMC8676388

[16] Galindo RJ, Beck RW, Scioscia MF et al. Glycemic monitoring and management in advanced chronic kidney disease. Endocr Rev 2020;41:756–74. 10.1210/endrev/bnaa017 32455432 PMC7366347

[17] Guthoff M, Wagner R, Vosseler D et al. Impact of end-stage renal disease on glucose metabolism—a matched cohort analysis. Nephrol Dial Transplant 2017;32:670–6. 10.1093/ndt/gfx018 28407130

[18] Coelho S, Rodrigues A. Hemoglobin A1c in patients on peritoneal dialysis: how should we interpret it? Therap Apheresis Dialysis 2014;18:375–82. 10.1111/1744-9987.12166 24571450

[19] Coelho S. What is the role of HbA1c in diabetic hemodialysis patients? Semin Dial 2016;29:19–23. 10.1111/sdi.12408 26138753

[20] Coelho S. Is the management of diabetes different in dialysis patients? Semin Dial 2018;31:367–76. 10.1111/sdi.12698 29659052

[21] Gillery P. HbA(1c) and biomarkers of diabetes mellitus in clinical chemistry and laboratory medicine: ten years after. Clin Chem Lab Med 2023;61:861–72. 10.1515/cclm-2022-0894 36239682

[22] Shima K, Chujo K, Yamada M et al. Lower value of glycated haemoglobin relative to glycaemic control in diabetic patients with end-stage renal disease not on haemodialysis. Ann Clin Biochem 2012;49:68–74. 10.1258/acb.2011.011161 22194360

[23] Freedman BI, Shenoy RN, Planer JA et al. Comparison of glycated albumin and hemoglobin A1c concentrations in diabetic subjects on peritoneal and hemodialysis. Perit Dial Int 2010;30:72–79. 10.3747/pdi.2008.00243 20056983

[24] Freedman BI, Shihabi ZK, Andries L et al. Relationship between assays of glycemia in diabetic subjects with advanced chronic kidney disease. Am J Nephrol 2010;31:375–9. 10.1159/000287561 20299782

[25] Peacock TP, Shihabi ZK, Bleyer AJ et al. Comparison of glycated albumin and hemoglobin A1c levels in diabetic subjects on hemodialysis. Kidney Int 2008;73:1062–8. 10.1038/ki.2008.25 18288102

[26] Jung M, Warren B, Grams M et al. Performance of non-traditional hyperglycemia biomarkers by chronic kidney disease status in older adults with diabetes: results from the Atherosclerosis Risk in Communities Study. J Diabetes 2018;10:276–85. 10.1111/1753-0407.12618 29055090 PMC5867205

[27] de Boer IH, Khunti K, Sadusky T et al. Diabetes management in chronic kidney disease: a consensus report by the American Diabetes Association (ADA) and Kidney Disease: Improving Global Outcomes (KDIGO). Diabetes Care 2022;45:3075–90. 10.2337/dci22-0027 36189689 PMC9870667

[28] Laghrib Y, Massart A, de Fijter JW et al. Pre-transplant HbA1c and risk of diabetes mellitus after kidney transplantation: a single center retrospective analysis. J Nephrol 2023;36:1921–9. 10.1007/s40620-023-01623-x 37039964

[29] Martin-Moreno PL, Shin H-S, Chandraker A. Obesity and post-transplant diabetes mellitus in kidney transplantation. J Clin Med 2021;10:2497. 10.3390/jcm10112497 34198724 PMC8201168

[30] Oniscu GC, Abramowicz D, Bolignano D et al. Management of obesity in kidney transplant candidates and recipients: a clinical practice guideline by the DESCARTES Working Group of ERA. Nephrol Dial Transplant 2021;37:i1–i15. 10.1093/ndt/gfab310 34788854 PMC8712154

[31] Chakkera HA, Chang YH, Ayub A et al. Validation of a pretransplant risk score for new-onset diabetes after kidney transplantation. Diabetes Care 2013;36:2881–6. 10.2337/dc13-0428 24009296 PMC3781551

[32] Torres A, Hernández D, Moreso F et al. Randomized controlled trial assessing the impact of tacrolimus versus cyclosporine on the incidence of posttransplant diabetes mellitus. Kidney Int Rep 2018;3:1304–15. 10.1016/j.ekir.2018.07.009 30450457 PMC6224662

[33] Vincenti F, Friman S, Scheuermann E et al. Results of an international, randomized trial comparing glucose metabolism disorders and outcome with cyclosporine versus tacrolimus. Am J Transplant 2007;7:1506–14. 10.1111/j.1600-6143.2007.01749.x 17359512

[34] Webster A, Woodroffe RC, Taylor RS et al. Tacrolimus versus cyclosporin as primary immunosuppression for kidney transplant recipients. Cochrane Database Syst Rev 2005;:Cd003961. 16235347 10.1002/14651858.CD003961.pub2

[35] Haller MC, Royuela A, Nagler EV et al. Steroid avoidance or withdrawal for kidney transplant recipients. Cochrane Database System Rev 2016;2016. 10.1002/14651858.CD005632.pub3 PMC8520739 27546100

[36] Stumpf J, Thomusch O, Opgenoorth M et al. Excellent efficacy and beneficial safety during observational 5-year follow-up of rapid steroid withdrawal after renal transplantation (Harmony FU study). Nephrol Dial Transplant 2023;39:141–50. 10.1093/ndt/gfad130 37391381 PMC10730794

[37] Ekberg H, Tedesco-Silva H, Demirbas A et al. Reduced exposure to calcineurin inhibitors in renal transplantation. N Engl J Med 2007;357:2562–75. 10.1056/NEJMoa067411 18094377

[38] Hellemans R, Bosmans JL, Abramowicz D. Early steroid withdrawal: a niche for anti-interleukin 2 receptor monoclonal antibodies? Nephrol Dial Transplant 2018;33:1083–7. 10.1093/ndt/gfy053 29596594

[39] Pernin V, Glyda M, Viklický O et al. Long-term prolonged-release tacrolimus-based immunosuppression in de novo kidney transplant recipients: 5-Y prospective follow-up of patients in the ADVANCE Study. Transplant Direct 2023;9:e1432. 10.1097/TXD.0000000000001432 36875940 PMC9977488

[40] Wissing KM, Abramowicz D, Weekers L et al. Prospective randomized study of conversion from tacrolimus to cyclosporine A to improve glucose metabolism in patients with posttransplant diabetes mellitus after renal transplantation. Am J Transplant 2018;18:1726–34. 10.1111/ajt.14665 29337426

[41] Hecking M, Haidinger M, Döller D et al. Early basal insulin therapy decreases new-onset diabetes after renal transplantation. J Am Soc Nephrol 2012;23:739–49. 10.1681/ASN.2011080835 22343119 PMC3312499

[42] Rodriguez-Rodriguez AE, Porrini E, Torres A. Beta-cell dysfunction unduced by tacrolimus: a way to explain type 2 diabetes? Int J Mol Sci 2021;22:10311. 10.3390/ijms221910311 34638652 PMC8509035

[43] Eide IA, Halden TAS, Hartmann A et al. Limitations of hemoglobin A1c for the diagnosis of posttransplant diabetes mellitus. Transplantation 2015;99:629–35. 10.1097/TP.0000000000000376 25162478

[44] Valderhaug TG, Jenssen T, Hartmann A et al. Fasting plasma glucose and glycosylated hemoglobin in the screening for diabetes mellitus after renal transplantation. Transplantation 2009;88:429–34. 10.1097/TP.0b013e3181af1f53 19667949

[45] Porrini EL, Díaz JM, Moreso F et al. Clinical evolution of post-transplant diabetes mellitus. Nephrol Dial Transplant 2016;31:495–505. 10.1093/ndt/gfv368 26538615

[46] Eide IA, Halden TA, Hartmann A et al. Mortality risk in post-transplantation diabetes mellitus based on glucose and HbA1c diagnostic criteria. Transpl Int 2016;29:568–78. 10.1111/tri.12757 26875590

[47] Kurnikowski A, Nordheim E, Schwaiger E et al. Criteria for prediabetes and posttransplant diabetes mellitus after kidney transplantation: a 2-year diagnostic accuracy study of participants from a randomized controlled trial. Am J Transplant 2022;22:2880–91. 10.1111/ajt.17187 36047565 PMC10087499

[48] Ussif AM, Åsberg A, Halden TAS et al. Validation of diagnostic utility of fasting plasma glucose and HbA1c in stable renal transplant recipients one year after transplantation. BMC Nephrol 2019;20:12. 10.1186/s12882-018-1171-3 30630438 PMC6327477

