# Supplementary material for: Current practices in prevention, screening, and treatment of diabetes in kidney transplant recipients: European survey highlights from the ERA DESCARTES Working Group
Source: Clin Kidney J. 2024 Dec 10;18(1):sfae367. doi: 10.1093/ckj/sfae367 (PMC11747291; doi:10.1093/ckj/sfae367)
Supplement: sfae367_Supplemental_Files [file sfae367_supplemental_files.zip › Supplemental S1 - full survey.docx]

Supplemental S1– the full survey

Introduction

Thank you in advance for taking the survey. This survey aims to get a better insight in the current practice in screening and prevention of post-transplant diabetes mellitus (PTDM) in Europe. We would like to start with some general introductory questions.

*Disclaimer: This survey should be completed by 1 nephrologist per transplant center. If the respondent wishes to be acknowledged as a collaborator in the publication that may result from this study, he/she can leave his/her name and affiliation in a designated field at the end of this survey. The results of the survey will be presented in an aggregated manner, to ensure that responses related to clinical management cannot be attributed to individual respondents.*

**Q1 Are you a nephrologist actively involved in the care of adult kidney transplant recipients in the first 3 months post-transplant?**

- No
- Yes

Display This Question:

If Q1 = No

Q1.1 We regret to inform you that this survey is designed for nephrologists actively involved in the care of adult kidney transplant recipients during their early post-transplant period. We thank you for your interest in this study and hope to work with you on future occasions.

**Q2 How many years of experience do you have in the care of kidney transplant recipients during their early post-transplant period?**

- ≤ 5 years
- 5 years

**Q3 Are you also certified as a specialist in endocrinology?**

- No
- Yes

**Q4 How many kidney transplants are performed in your center yearly?**

- <25
- 25-49
- 50-100
- >100

**Q5 In which country do you currently work?**

▼ Albania ... United Kingdom of Great Britain and Northern Ireland

Explanation The next questions relate to your approach to screening and prevention of post-transplant diabetes mellitus (PTDM).

Please note that the questions will appear in the following order:

1. During the pre-transplant work-up
2. At the day of transplantation
3. During the early post-transplant phase (≤ 45days) 
4. After 45 days post-transplant

Phase 1 The next questions focus on the **pre-transplant work-up.**

**Q6 Do you routinely screen for diabetes mellitus in the pretransplant work-up?**

- No
- Yes

Display This Question:

If Q6 = Yes

**Q6.1 How do you routinely screen for diabetes mellitus in the pretransplant work-up?
(more than one answer is possible)**

- Random (non-fasting) glycemia
- Fasting glycemia
- Oral glucose tolerance test
- HbA1c

Display This Question:

If Q6 = yes

**Q6.2 Since the time between the pre-transplantation work-up and the actual transplantation can be several years, do you repeat the diabetes screening during the waiting period?**

- No
- Sometimes
- (At least) annually

**Q7 Do you record the familial history of diabetes mellitus in the patients’ medical file?**

- No
- Sometimes
- Yes, always

**Q8 Do you have a specific weight-management program for obese transplant candidates?**

- No
- Yes

Display This Question:

If Q8 = yes

**Q8.1 Which options are included in your weight-management program?
(more than one answer is possible)**

- Diet and intensive follow up by a dietician
- Tailored exercise program
- GLP-1 analogues
- Bariatric surgery

Phase 2 The following questions focus **on the day of transplantation.**

Please note that we are specifically interested in your approach to **screening and prevention of PTDM** (*not your management of patients with pre-existing diabetes mellitus*).

**Q9 How do you routinely screen for pre-existing diabetes mellitus on the day of transplantation?
(more than one option is possible)**

- Random glycemia on admission (generally non-fasting)
- Fasting glycemia **only in living donor** transplantation
- Fasting glycemia **in both living and deceased donor** transplantation
- Oral glucose tolerance test  **only in living donor** transplantation
- Oral glucose tolerance test  **in both living and deceased** transplantation
- HbA1c

**Q10 Do you have, in advance, a differentiated post-transplant management plan (e.g. choice of immunosuppression, intensity of glucose monitoring) depending on your perceived PTDM risk on the day of transplantation?**

- No
- Yes

Display This Question:

If Q10 = yes

**Q10.1 Which items do you include in your risk assessment for PTDM on the day of transplantation?
 (more than one option is possible)**

- Age
- BMI
- Waist circumference
- Waist-hip ratio
- Family history
- Fasting glycemia
- HbA1c
- Triglyceridemia
- Findrisc score_*_ *_(Lindström J, Tuomilehto J. The Diabetes Risk Score. Diabetes Care. 2003;26(3):725-731)_* _(7)_
- Score by Chakkera et al_.**_ *_(Chakkera H, et al. Pretransplant Risk Score for New-Onset Diabetes After Kidney Transplantation. Diabetes Care. 2011;34(10):2141-2145.)_* _(15)_
- Other (17)

Display This Question:

If Q10.1 = other

**Please specify other Please specify other:**

________________________________________________________________

***Findrisc score included parameters:** age, BMI, use of blood pressure medication, history of high blood glucose, physical activity, daily consumption of vegetables, fruits, or berries and/or family history of diabetes
****Chakkera included parameters:**age, BMI, history of high blood glucose, family history of diabetes, planned maintenance therapy with corticosteroids, use of gout medicine and/or history of high triglycerides 

Display This Question:

If Q10 = yes

**Q11 Do you routinely consider a different immunosuppressive strategy on the day of transplantation in patients deemed at higher risk of PTDM?**

- No, patients with or without an increased risk of PTDM start with a similar immunosuppressive regimen
- Yes, I consider one or more of the following alternative strategies (more than one answer possible):
  - Cyclosporin instead of tacrolimus
  - Calcineurin inhibitor-free regimen with belatacept
  - Calcineurin inhibitor-free regimen with mTOR inhibitor
  - Calcineurin inhibitor plus mTOR inhibitor
  - Very early steroid withdrawal (≤ 1 week post-transplant)
  - Intermediate-early steroid withdrawal (between 1 week – 3 months post-transplant)
  - Steroidal withdrawal after 3 months

Phase 3 The next questions focus on the **immediate post-transplant period (≤ 45 days)**
Please note that we refer to hyperglycemia in the first 45 days as **early post-transplant hyperglycemia**, while **the diagnosis of PTDM can only be made after 45 days post-transplantation**.
*(Based on consensus Sharif A et al, Proceedings From an International Consensus Meeting on*

*Posttransplantation Diabetes Mellitus: Recommendations and Future Directions. American Journal of Transplantation 2014 Vol. 14 Issue 9 Pages 1992-2000  )*

**Q12 In which patients do you perform glucose day profile monitoring during hospitalization after transplantation?**

- In none of the patients
- In the high-risk patients
- In all patients

**Q13 How long do you continue glucose day profile monitoring during hospitalization after transplantation?**

- Routinely during the first 1-3 days (and continued in those who develop hyperglycemia)
- Routinely during the first 4-7 days (and continued in those who develop hyperglycemia)
- Routinely more than 7 days

**Q14 Do you routinely apply very tight glycaemic control during the early post-transplant period with the use of long-acting insulin therapy once the postoperative afternoon glucose value exceeds 140mg/dL (7.8mmol/L), targeting a pre-dinner glycemia of 110 mg/dl (6.1mmol/L)  post-transplant, such as advocated in the study by Schwaiger et al?**
 *(Schwaiger E, et al. Early Postoperative Basal Insulin Therapy versus Standard of Care for the Prevention of Diabetes Mellitus after Kidney Transplantation: A Multicenter Randomized Trial. Journal of the American Society of Nephrology. 2021;32(8):2083-2098.)*

- No
- Yes, in all patients
- Yes, in selected patients deemed at high risk of developing PTDM
- Yes, but with slightly different glucose target levels than those advocated by Schwaiger et al.

**Q15 Do you organize home blood glucose monitoring during the early post-transplant phase (≤ 45 days)?**

- No
- Yes, on a case per case basis
- Yes, always

**Q16 Do you refer patients with early post-transplant hyperglycemia (≤ 45 days) to an endocrinologist?**

- No
- Yes, on a case per case basis
- Yes, always

**Q17 Do you change immunosuppression in patients who develop early post-transplant hyperglycemia (≤ 45 days)?**

- No
- Yes, on a case per case basis
- Yes, this is a routine procedure in patients at standard rejection risk

Skip To: Q17.2.1 If Q17 = no

Display This Question:

If Q17 = Yes…

**Q17.1 Do you consider the withdrawal of corticosteroids in patients who develop early post-transplant hyperglycemia (≤ 45 days)?**

- No
- Yes

Display This Question:

If Q17.1 = 2

**Q17.1.1 When do you consider the withdrawal of corticosteroids? 
(more than one option is possible)**

Very early steroid withdrawal (≤ 1 week post-transplant) (1)

Intermediate-early steroid withdrawal (between 1 week – 3 months post-transplant) (2)

Steroidal withdrawal after 3 months (3)

Display This Question:

If Q17 = Yes

**Q17.2 Do you consider changing, avoiding or minimizing calcineurin inhibitors, in patients who develop early post-transplant hyperglycemia in the first 45 days?**

- No
- Yes

Display This Question:

If Q17.2 = yes

**Q17.2.1 What do you consider for patients who develop early post-transplant hyperglycemia in the first 45 days, who are on a standard regimen with calcineurin-inhibitors and mycophenolate? (more than one option possible)**

- Reduction of calcineurin dose
- Switch from tacrolimus to cyclosporine
- Switch from calcineurin inhibitor to belatacept
- Switch from calcineurin inhibitor to mTOR inhibitor
- Switch to low-dose calcineurin inhibitor plus mTOR inhibitor

**Q18 Do you use antidiabetic drugs other than insulin in hyperglycemic patients during the early post-transplant period (≤ 45 days) ?**

- No
- Yes

Display This Question:

If Q18 = yes

**Q18.1 Which antidiabetic drugs other than insulin do you consider in the early post-transplant period (≤ 45 days)?**(more than one option is possible)

- Metformin
- Sulfonylurea or glinides
- DPP-4 inhibitors
- GLP-1 analogues
- SGLT-2 inhibitors

Phase 4 The next questions focus on the period **after 45 days post-transplantation.**
From 45 days on, PTDM can formally be diagnosed.
*(Based on consensus Sharif A et al, Proceedings From an International Consensus Meeting on Posttransplantation Diabetes Mellitus: Recommendations and Future Directions. American Journal of Transplantation 2014 Vol. 14 Issue 9 Pages 1992-2000)*

**Q19 Do you have a defined protocol to screen for PTDM between 45 days and 6 months?**

- No
- Yes

Display This Question:

If Q19 = yes

**Q20 What is included in your protocol to screen for PTDM between 45 days and 6 months?
(More than one option is possible)**

- Fasting glycemia
- Random (non-fasting) glycemia
- Oral glucose tolerance test
- HbA1c
- Home glucose measurements

**Q21 Do you have a defined protocol to screen for PTDM annually?**

- No
- Yes

Display This Question:

If Q21 = yes

**Q21.1 What is included in your protocol to screen for PTDM between annually?
 (More than one option is possible)**

- Fasting glycemia
- Random (non-fasting) glycemia
- Oral glucose tolerance test
- HbA1c
- Home glucose measurements

**Q22 Do you refer patients who are diagnosed with PTDM after 45 days to an endocrinologist?**

- No
- Yes, on a case per case basis
- Yes, this is standard procedure

**Q23 Do you change immunosuppression in patient who are diagnosed with PTDM after 45 days post-transplantation?**

- No
- Yes, on a case per case basis
- Yes, this is a standard procedure

Skip To: End of Block If Q23 = No

Display This Question:

If Q23 = Yes

**Q23.1 Do you consider the withdrawal of corticosteroids after 45 days post-transplantation?**

- No
- Yes

Display This Question:

If Q23.1 = Yes

**Q23.1.1 When do you consider the withdrawal of corticosteroids? 
(more than one option is possible)**Intermediate-early steroid withdrawal (between 6 weeks – 3 months post-transplant)

Steroidal withdrawal after 3 months

Display This Question:

If Q23 = yes

**Q23.2 Do you consider changing, avoiding or minimizing calcineurin inhibitors in patients who develop PTDM after 45 days post-transplant?**

- No
- Yes

Display This Question:

If Q23.2 = Yes

**Q23.2.1 What do you consider for patients who develop PTDM, who are on a standard regimen with calcineurin-inhibitors and mycophenolate? (more than one option possible)**

- Reduction of calcineurin dose
- Switch from tacrolimus to cyclosporine
- Switch from calcineurin inhibitor to belatacept
- Switch from calcineurin inhibitor to mTOR inhibitor
- Switch to low-dose calcineurin inhibitor plus mTOR inhibitor

**Q24 Which antidiabetic drugs other than insulin do you consider in patients who have developed PTDM (after 45 days post-transplant)**

- Metformin
- Sulfonylurea or glinides
- DPP-4 inhibitors
- GLP-1 analogues
- SGLT-2 inhibitors

**Q25 Finally, we would like to get an idea on the (perceived) incidence of PTDM during the first year posttransplant in your center.
Do you know the current 1-year incidence of PTDM (excluding patients with pre-existing diabetes mellitus) at your center?**

- No
- Yes

Display This Question:

If Q25 = Yes

**Q25.1 Please provide the current 1-year incidence of PTDM (as a percentage) at your center (excluding patients with pre-existing diabetes mellitus):**

Display This Question:

If Q25 = No

**Q25.2 Please provide your best guess about the current 1-year incidence of PTDM (as a percentage) at your center (excluding patients with pre-existing diabetes mellitus):**

**Q26 Would you like to be registered as a contributor to this study, and listed as a collaborator in the publication that may result from this survey?**

- No
- Yes

Display This Question:

If Q26 = yes

**Q26.1 Name:**

Display This Question:

If Q26 = yes

**Q26.2 Affiliation (transplant center where you work):**
