# Supplementary material for: Current practices in prevention, screening, and treatment of diabetes in kidney transplant recipients: European survey highlights from the ERA DESCARTES Working Group
Source: Clin Kidney J. 2024 Dec 10;18(1):sfae367. doi: 10.1093/ckj/sfae367 (PMC11747291; doi:10.1093/ckj/sfae367)

Supplemental S2 – additional Figures

The survey conducted among the participants inquired about their knowledge of PTDM incidence at 12 months post-transplantation. The supplementary figures display the reported incidence, whether known or estimated.

**Figure 1: known PTDM incidence at 12 months post-transplantation**


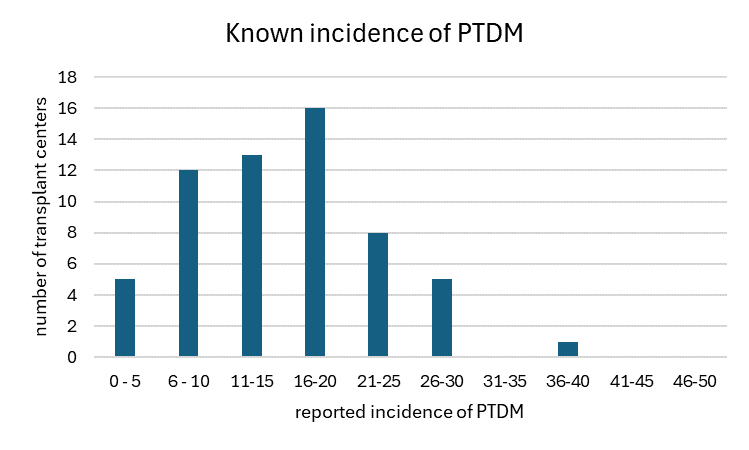


**Figure 2: estimated PTM incidence at 12 months post-transplantation**


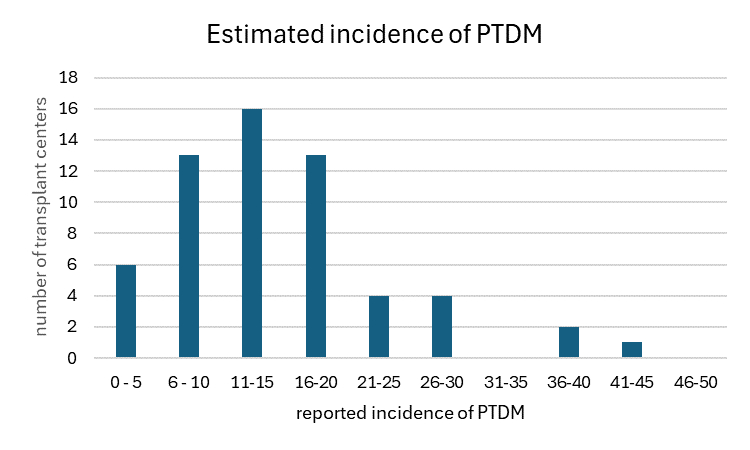

Supplement: sfae367_Supplemental_Files [file sfae367_supplemental_files.zip › Supplemental S2 - additional figures.docx]
