# Supplementary material for: Current practices in prevention, screening, and treatment of diabetes in kidney transplant recipients: European survey highlights from the ERA DESCARTES Working Group
Source: Clin Kidney J. 2024 Dec 10;18(1):sfae367. doi: 10.1093/ckj/sfae367 (PMC11747291; doi:10.1093/ckj/sfae367)
Supplement: sfae367_Supplemental_Files [file sfae367_supplemental_files.zip › Supplemental S3 - difference between small and large centers.docx]

Supplemental S3 - difference between small and large transplant centres

Transplant centres with less than 50 transplantations annually were considered small (S) and ≥ 50 were considered large (L).

**Table 1: Screening for diabetes mellitus pre-transplantation, risk assessment for PTDM, and management of hyperglycaemia peri-transplantation.**

| **Pre-transplant workup** |
| --- |

| 1. ***Do you routinely screen for diabetes mellitus in the pretransplant work-up? Yes,***   **S** 35/41 (85%)  **L** 69/80 (86%) |
| --- |
| **If yes:** |
| *1.1. How do you routinely screen for diabetes mellitus in the pretransplant work-up?*  *(more than one answer possible)* |
| \| - HbA1c \| ***S*** *28/35 (80%)*  ***L*** *62/69 (90%)* \| - Random glycemia \| **S** 9/35 (26%)  **L** 17/69 (25%) \| \| --- \| --- \| --- \| --- \| \| - Fasting glycemia \| ***S*** *28/35 (80%)*  ***L*** *45/69 (65%)* \| - OGTT \| **S** 3/35 (9%)  **L** 11/69 (16%) \| |
| *1.2. Since the time between the pre-transplantation work-up and the actual transplantation can be several years, do you repeat the diabetes screening during the waiting period* |
| \| - Yes (at least) annually \| **S** 28/35 (80%)  **L** 31/69 (45%) \| \| --- \| --- \| \| - Sometimes \| **S** 6/35 (17%)  **L** 28/69 (41%) \| \| - No \| **S** 1 /104 (1%)  **L** 10/69 (14%) \| |

| 1. ***Do you have a specific weight-management program for obese transplant candidates?* Yes**,   ***S*** *21/41 (51%)*  ***L*** *43/80 (54%)* |
| --- |
| **If yes:** |
| *2.1. Which options are included in your weight-management program?*  *(more than one answer possible)* |
| \| - Diet and intensive follow-up by a dietician \| **S** 20/21 (95%)  **L** 42/43 (98%) \| - Exercise program \| **S** 8/21 (38%)  **L** 20/43 (47%) \| \| --- \| --- \| --- \| --- \| \| - Bariatric surgery \| **S** 10/21 (48%)  **L** 34/43 (79%) \| - GLP-1 analogue \| **S** 6/21 (29%)  **L** 22/43 (51%) \| |

| ***3. Do you record the family history of diabetes mellitus in the patient’s medical file?*** |
| --- |
| *2.1. Which options are included in your weight-management program?* |
| \| - Yes always \| **S** 34/41 (83%)  **L** 41/80 (51%) \| \| --- \| --- \| \| - Sometimes \| **S** 5/41 (12%)  **L** 32/80 (40%) \| \| - No \| **S** 2/41 (5%)  **L** 7/80 (9%) \| |

| **Transplantation** |
| --- |

| ***4. How do you routinely screen for pre-existing diabetes mellitus on the day of transplantation?***  *(more than one answer possible)* |
| --- |
| *2.1. Which options are included in your weight-management program?* |
| \| - HbA1c \| **S** 20/41 (49%)  **L** 53/80 (66%) \| \| --- \| --- \| \| - Random glycaemia \| **S** 24/41 (59%)  **L** 49/80 (61%) \| \| - Fasting glycaemia in both living and deceased donor recipients \| **S** 19/41 (46%)  **L** 33/80 (41%) \| \| - Fasting glycaemia only in living donor recipients \| **S** 10/41 (24%)  **L** 14/80 (18%) \| \| - OGTT only in living donor recipients \| **S** 2/41 (5%)  **L** 5/80 (6%) \| \| - OGTT in both living and deceased donor recipients \| **S** 1/41 (2%)  **L** 2/80 (3%) \| |

| ***5. Do you have a differentiated post-transplant management plan (e.g., choice of immunosuppression, intensity of glucose monitoring) in advance based on your perceived PTDM risk on the day of transplantation? Yes,***  **S** 19/41 (46%)  **L** 29/80 (36%) |
| --- |
| **If yes:** |
| *5.1. Which items do you include in your risk assessment for PTDM on the day of transplantation?*  *(more than one answer possible)* |
| \| **Demographics** \|  \| **Lab values** \|  \| \| --- \| --- \| --- \| --- \| \| - Age \| **S** 11/19 (58%)  **L** 23/29 (79%) \| - Triglyceridemia \| **S** 4/19 (21%)  **L** 12/29 (41%) \| \| - Family history \| **S** 14/19 (74%)  **L** 21/29 (72%) \| - HbA1c \| **S** 18/19 (95%)  **L** 24/29 (83%) \| \| - BMI \| **S** 18/19 §(95%)  **L** 26/29 (90%) \| - Fasting glycaemia \| **S** 17/19 (22%)  **L** 19/29 (66%) \| \| - Waist circumference \| **S** 4/19 (21%)  **L** 6/29 (21%) \| **Models** \|  \| \| - Waist/hip ratio \| **S** 3/19 (16%)  **L** 1/29 (3%) \| Chakkera model \| **S** 0/19 (0%)  **L** 2/29 (7%) \| \|  \|  \| Findrisc model \| **S** 1/19 (5%)  **L** 0/29 (0%) \| |
| *5.2. Do you routinely consider a different immunosuppressive strategy on the day of transplantation in patients deemed at higher risk of PTDM? Yes,*  **S** 7/19 (37%)  **L** 16/29 (55%) |
| **If yes:** |
| 5.2.1. Yes, I consider one or more alternative strategies  *(more than one answer possible)* |
| \| **CNI regimen** \|  \| **Stop corticosteroids** \|  \| \| --- \| --- \| --- \| --- \| \| - Cyc instead of Tac \| **S** 2/7 (29%)  **L**10/16 (63%) \| - First week \| **S** 0/7 (0%)  **L** 2 /16 (13%) \| \| - mTOR instead of CNI \| **S** 1/7 (14%)  **L** 0/16 (0%) \| - W1-3m \| **S** 2/7 (29%)  **L** 7/16 (44%) \| \| - mTOR + lower dose of CNI \| **S** 0/7 (0%)  **L** 1/16 (6%) \| - >3 months \| **S** 3/7 (43%)  **L** 4/16 (25%) \| \| - Belatacept instead of CNI \| **S** 0/7 (0%)  **L**1/16 (6%) \|  \|  \| |

| ***6. Do you routinely apply very tight glycaemic control during the early post-transplant period with the use of long-acting insulin therapy once the postoperative afternoon glucose value exceeds 140 mg/dL (7.8 mmol/L), targeting a pre-dinner glycemia of 110 mg/dl (6.1 mmol/L) post-transplant, such as advocated in the study by Schwaiger et al.?*** |
| --- |
| \| - No \| **S** 18/41 (44%)  **L** 40/80 (50%) \| \| --- \| --- \| \| - Yes, but with slightly different glucose target levels than those advocated by Schwaiger et al.(1) \| **S** 10/41 (24%)  **L** 13/80 (16%) \| \| - Yes, in all patients \| **S** 5/41 (12%)  **L**16/80 (20%) \| \| - Yes, in selected patients deemed at high risk of developing PTDM \| **S** 8/41 (20%)  **L** 11/80 (14%) \| |

| ***7. How long do you continue glucose day profile monitoring during hospitalization after transplantation?*** |
| --- |
| \| - Routinely during the first 4-7 days (and continued in those who develop hyperglycaemia \| **S** 14/41 (34%)  **L** 41/80 (51%) \| \| --- \| --- \| \| - Routinely, more than 7 days \| **S** 17/41 (42%)  **L** 24/80 (30%) \| \| - Routinely during the first 1-3 days (and continued in those who develop hyperglycaemia) \| **S** 10/41 (24%)  **L** 15/80 (19%) \| |

**Table 2: Post-transplant screening and management of hyperglycaemia.**

| **Early post-transplant phase < 45 days** |
| --- |

| ***1. Do you organize home blood glucose monitoring during the early post-transplant phase (≤ 45 days)?*** |
| --- |
| \| - Yes, on a case-per-case basis \| **S** 27/41 (66%)  **L** 58/80 (73%) \| \| --- \| --- \| \| - No \| **S** 9/41 (22%)  **L** 19/80 (24%) \| \| - Yes, always \| **S** 5/41 (12%)  **L** 3/80 (4%) \| |

| ***2. Do you refer patients with early post-transplant hyperglycaemia (≤ 45 days) to an endocrinologist?*** |
| --- |
| \| - Yes, on a case-per case basis \| **S** 21/41 (51%)  **L** 42/80 (53%) \| \| --- \| --- \| \| - Yes, always \| **S** 14/41 (34%)  **L** 19/80 (24%) \| \| - No \| **S** 6/41 (15%)  **L** 19/80 (24%) \| |

| ***3. Do you change immunosuppression in patients who develop early post-transplant hyperglycaemia (≤ 45 days)?*** |
| --- |
| \| - No \| **S** 27/41 (66%)  **L** 45/80 (56%) \| \| --- \| --- \| \| - Yes, on a case-per case basis \| **S** 14/41 (34%)  **L** 33/80 (41%) \| \| - Yes, routinely in patients with standard risk for PTDM \| **S** 0/41 (0%  **L** 2/80 (3%) \| |
| *3.1. Do you consider the withdrawal of corticosteroids in patients who develop early post-transplant hyper-glycemia (≤ 45 days)? Yes,*  **S** 7/14 (50%)  **L** 20/35 (57%) |
| **If yes:** |
| 3.1.1. When do you consider the withdrawal of corticosteroids?  *(more than one answer possible)* |
| \| - Fist week \| **S** 1/7 (14%)  **L** 5/20 (25%) \| \| --- \| --- \| \| - Week 1 – 3 months \| **S** 3/7 (43%)  **L** 10/20 (50%) \| \| - > 3 months \| **S** 4/7 (57%)  **L** 8/20 (40%) \| |
| *3.2. Do you consider changing, avoiding, or minimizing calcineurin inhibitors in patients who develop early post-transplant hyperglycaemia in the first 45 days? Yes,*  **S** 6/14  **L** 12/35 |
| **If yes:** |
| 3.2.1. What do you consider for patients who develop early post-transplant hyperglycaemia in the first 45 days and are on a standard regimen with calcineurin inhibitors and mycophenolate?  *(more than one answer possible)* |
| \| - Switch to CsA instead of Tac \| **S** 4/6 (67%)  **L** 9/12 (75%) \| \| --- \| --- \| \| - Reduction CNI dose \| **S** 2/6 (33%)  **L** 6/12 (50%) \| \| - Switch to mTOR + low dose CNI \| **S** 3/6 (50%)  **L** 1/12 (8%) \| \| - Switch to Belatacept instead of CNI \| **S** 0/6 (0%)  **L** 2/12 (17%) \| \| - Switch to mTOR instead of CNI \| **S** 1/6 (13%)  **L** 0/12 (0%) \| |

| ***4. Do you use antidiabetic drugs other than insulin in hyperglycaemia patients during the early post-transplant period (≤ 45 days)? Yes,***  **S** 27/41 (66%)  **L** 58/80 (73%) |
| --- |
| *4.1. Which antidiabetic drugs other than insulin do you consider in the early post-transplant period (≤ 45 days)?*  (more than one answer possible) |
| \| - DPP-4 inhibitors \| **S** 19/27 (70%)  **L** 33/58 (57%) \| \| --- \| --- \| \| - Metformin \| **S** 14/27 (52%)  **L** 28/58 (48%) \| \| - Sulfonylurea or glinides \| **S** 7/27 (26%)  **L** 31/58 (53%) \| \| - SGLT2-inhibitors \| **S** 9/27 (33%)  **L**12/58 (21%) \| \| - GLP-1 analogues \| **S** 4/27 (15%)  **L** 13/58 (22%) \| |

| **Post-transplant phase > 45 days** |
| --- |

| ***5. Do you have a defined protocol to screen for PTDM between 45 days and six months? Yes,***  S 27/41 (66%)  L 55/80 (69%) |
| --- |
| **If yes:** |
| *5.1. What is included in your protocol to screen for PTDM between 45 days and six months?*  (more than one answer possible) |
| \| - Fasting glycaemia \| **S** 25/27 (93%)  **L** 45/55 (82%) \| \| --- \| --- \| \| - HbA1c \| **S** 23/27 (85%)  **L** 47/55 (85%) \| \| - Home glucose measurements \| **S** 10/27 (37%)  **L** 9/55 (16%) \| \| - Random glycaemia \| **S** 5/27 (19%)  **L** 14/55 (25%) \| \| - OGTT \| **S** 5/27 (19%)  **L** 3/55 (5%) \| |

| ***6. Do you have a defined protocol to screen for PTDM annually? Yes,***  **S** 31/41 (76%)  **L** 59/80 (74%) |
| --- |
| **If yes:** |
| *6.1. What is included in your protocol to screen for PTDM annually?*  (more than one answer possible) |
| \| - Fasting glycaemia \| **S** 28/31 (90%)  **L** 47/59 (80%) \| \| --- \| --- \| \| - HbA1c \| **S** 29/31 (94%)  **L** 56/59 (95%) \| \| - Random glycaemia \| **S** 5/31 (16%)  **L** 14/59 (24%) \| \| - Home glucose measurements \| **S** 7/31 (23%)  **L** 6 /59 (10%) \| \| - OGTT \| **S** 2/31(6%)  **L** 3/59 (5%) \| |

| ***7. Do you refer patients diagnosed with PTDM after 45 days to an endocrinologist?*** |
| --- |
| \| - Yes, on a case-per-case basis (%) \| **S**19/41 (46%)  **L** 39/80 (49%) \| \| --- \| --- \| \| - Yes, this is the standard procedure (%) \| **S** 20/41 (49%)  **L** 28/80 (35%) \| \| - No (%) \| **S** 2/41 (5%)  **L** 13/80 (16%) \| |

| ***8. Do you change immunosuppression in patients diagnosed with PTDM after 45 days post-transplantation?*** |
| --- |
| \| - Yes, on a case-per-case basis (%) \| **S** 25/41 (61%)  **L** 44/80 (55%) \| \| --- \| --- \| \| - No (%) \| **S** 16/41(39%)  **L** 35/80 (44%) \| \| - Yes, this is the standard procedure (%) \| **S** 0/41 (0%)  **L** 1/80 (1%) \| |
| **If yes:** |
| *8.1. Do you consider the withdrawal of corticosteroids after 45 days post-transplantation? Yes,*  S 12/25 (48%)  L 24/45 (53%), when:  (more than one answer possible) |
| \| **Stop corticosteroids** \|  \| \| --- \| --- \| \| - Steroidal withdrawal after three months \| **S** 11/12 (92%)  **L** 17/24 (71%) \| \| - Intermediate-early steroid withdrawal (between 6 weeks – 3 months post-transplant) \| **S** 2/12 (17%)  **L** 12/24 (50%) \| |
| *8.2. Do you consider changing, avoiding, or minimizing calcineurin inhibitors in patients who develop PTDM after 45 days post-transplantation? Yes,*  **S** 16/25 (64%)  **L** 27/45 (60%)  (more than one answer possible) |
| \| **CNI regimen** \|  \| \| --- \| --- \| \| - Switch to CsA instead of Tac \| **S** 7/16 (44%)  **L** 19/27 (70%) \| \| - Reduction CNI dose \| **S** 7/16 (44%)  **L** 17/27 (63%) \| \| - Switch to mTOR + low dose CNI \| **S** 7/16 (44%)  **L** 6/27 (22%) \| \| - Switch to mTOR instead of CNI \| **S** 6/16 (38%)  **L** 2/27 (7%) \| \| - Switch to Belatacept instead of CNI \| **S** 0/16 (0%)  **L** 3/27 (11%) \| |

| ***9. Which antidiabetic drugs other than insulin do you consider in patients who have developed PTDM (after 45 days post-transplant)?***  (more than one answer possible) |
| --- |
| \| - Metformin \| **S** 16/25 (64%)  **L** 36/45 (80%) \| \| --- \| --- \| \| - DPP-4 inhibitors \| **S** 17/25 (68%)  **L** 32/45 (71%) \| \| - SGLT2-inhibitors \| **S** 13/25 (52%)  **L** 35/45 (78%) \| \| - GLP-1 analogues \| **S** 11/25 (44%)  **L** 34/45 (76%) \| \| - Sulfonylurea or glinides \| **S** 7/25 (28%)  **L** 21/45(47%) \| |
